# Advances in protein microarray–based proteomics for gastric cancer applications in biomarker discovery, molecular subtyping, and precision oncology

**DOI:** 10.3389/fonc.2026.1907652

**Published:** 2026-09-04

**Authors:** Panning Wang, Yu-Lin Xiao, Shuhong Luo, Hua Dong, Ruo-Pan Huang

**Affiliations:** 1 Department of Biomedical Engineering, School of Materials Science and Engineering, South China University of Technology, Guangzhou, Guangdong, China; 2 Raybiotech Co., Ltd., Guangzhou, Guangdong, China; 3 South China Biotech Research Center, Guangzhou, Guangdong, China; 4 Department of Urology, The Third Affiliated Hospital of Sun Yat-sen University, Guangzhou, Guangdong, China; 5 RayBiotech Inc., Peachtree Corners, GA, United States; 6 National Engineering Research Center for Tissue Restoration and Reconstruction (NERC-TRR), Guangzhou, Guangdong, China; 7 Affiliated Cancer Hospital and Institute of Guangzhou Medical University, Guangzhou, Guangdong, China

**Keywords:** biomarker discovery, early diagnosis, gastric cancer, molecular subtyping, prognosis, protein microarray, proteomics

## Abstract

Gastric cancer remains a major cause of cancer-related mortality worldwide, largely due to late diagnosis and pronounced molecular heterogeneity. Although genomic and transcriptomic studies have advanced understanding of gastric tumor biology, they do not fully capture dynamic changes in protein expression, post-translational modifications, and signaling activity that directly regulate cellular function. High-throughput proteomic technologies, particularly protein microarrays, have emerged as powerful platforms for systematic protein profiling in complex biological samples. These technologies enable the simultaneous quantification of hundreds to thousands of proteins with high sensitivity and minimal sample requirements, supporting both discovery and translational research. Recent applications of protein microarrays in gastric cancer include biomarker discovery for early diagnosis, molecular subtyping, characterization of the tumor microenvironment, and assessment of therapeutic response. Multi-protein biomarker panels and integration with computational approaches have demonstrated improved diagnostic performance compared with conventional single-marker assays. In addition, proteomic profiling has provided insights into key signaling pathways and immune-related mechanisms underlying tumor progression and drug resistance. This review summarizes the principles and major formats of protein microarray technologies and highlights their applications in gastric cancer. Current challenges and future directions for clinical translation are also discussed.

## 1 Introduction

Gastric cancer (GC) remains one of the leading malignancies in terms of both incidence and mortality worldwide, posing a heavy burden on global public health (1–3). According to GLOBOCAN 2024 data, there were an estimated 980, 000 new cases and 642, 000 deaths globally, accounting for 4.7% and 6.6% of all new cancer cases and cancer deaths, respectively, making gastric cancer the fifth most common malignancy and the fifth leading cause of cancer-related deaths worldwide (1). Despite notable advances in endoscopic screening, surgical techniques, and targeted therapies, the overall prognosis of GC remains poor. This is largely attributable to the asymptomatic nature of early-stage disease, rapid tumor progression, pronounced molecular heterogeneity, and the lack of sensitive and specific biomarkers for early detection and prognostic assessment. Consequently, most patients are diagnosed at advanced stages, with a 5-year survival rate of less than 30% (2, 4–6).

Currently, GC diagnosis primarily relies on endoscopy and histopathological evaluation, supplemented by imaging modalities such as computed tomography (CT), magnetic resonance imaging (MRI), and endoscopic ultrasound. However, these approaches are invasive, costly, and not well suited for large-scale population screening (7–10). In addition, widely used serum tumor markers, including carcinoembryonic antigen (CEA), carbohydrate antigen 19-9 (CA19-9), and carbohydrate antigen 72-4 (CA72-4), exhibit limited sensitivity as adjunctive diagnostic biomarkers for GC. CEA and CA19–9 are tumor-associated glycoproteins that lack organ specificity; they can be elevated in other malignancies (e.g., colorectal, pancreatic) and benign conditions (e.g., pancreatitis, biliary obstruction), leading to false positives (11–14). Mechanistically, CEA functions as an intercellular adhesion molecule that participates in tumor invasion and metastasis through homophilic and heterophilic interactions (15, 16). CA19-9 (sialyl Lewis a) promotes hematogenous dissemination by binding to E-selectin and P-selectin on vascular endothelial cells (17, 18). These biological properties explain why both markers tend to become significantly elevated only when tumors have progressed to advanced stages with larger tumor burdens. Systematic review data demonstrate that the positivity rates of CEA and CA19–9 for stage I GC are only 13.7% and 9.0%, respectively, rising to 39.5% and 44.7% only at stage IV (11). Similarly, CA72–4 also exhibits a low positivity rate in early-stage GC (approximately 12.0% at stage I) (11), and multiple multivariate analyses have shown that its independent prognostic value is often superseded after adjustment for CA19–9 and TNM stage; not all studies support it as an independent predictor of recurrence (19, 20). CA125, while demonstrating promising predictive value for peritoneal metastasis in GC (at a cutoff of >22.75 U/mL, AUC: 0.771, sensitivity: 73.5%, specificity: 77.7%) (21, 22), shows extremely low overall diagnostic sensitivity as a broad-spectrum screening marker (ranging from 6% to 29.2% in some studies), far from meeting the requirements for routine early GC screening, Nevertheless, it remains a useful adjunctive marker for further evaluation in selected patients, particularly when peritoneal involvement is suspected (19, 23). In summary, the limited sensitivity of these conventional markers renders them insufficient for precise early screening and diagnosis of GC. To overcome these limitations, current research directions include: improving the performance of existing markers through combined detection strategies (24, 25), and optimized cut-off values (26); discovering novel serum biomarkers such as multi-protein panels (23, 27, 28) and autoantibody signatures (29–31); and constructing multi-omics integrated diagnostic models that incorporate proteomic, genomic, and clinical data (32–34). These strategies collectively aim to enhance early detection and risk stratification for GC.

Therefore, there is an urgent need to develop highly sensitive and specific noninvasive or minimally invasive biomarkers, along with robust, system-level molecular profiling platforms. Such advances are essential to enable early diagnosis, precise molecular stratification, and real-time therapeutic monitoring, ultimately improving clinical outcomes for patients with gastric cancer (35–38).

With the rapid advancement of high-throughput sequencing technologies, genomic and transcriptomic studies have provided critical insights into molecular subtypes and driver gene alterations in gastric cancer (GC) (39–41). However, mRNA expression levels do not necessarily correlate with protein abundance, post-translational modifications (PTMs), or signaling pathway activity. As the direct effectors of cellular function, proteins more accurately reflect the real-time physiological state of cells and the dynamic progression of disease (42–44). Accordingly, proteomics-based systematic profiling of protein expression and signaling networks has emerged as an essential approach for elucidating tumor biology and identifying clinically relevant biomarkers. High-throughput proteomics enables the identification of differentially expressed proteins associated with tumor initiation and progression, thereby facilitating biomarker discovery, therapeutic target identification, and mechanistic investigation, while also supporting clinical translation (45–50).

Mass spectrometry (MS)-based proteomics is currently the most widely used approach in the discovery phase, owing to its broad proteome coverage and high analytical depth (51–54). However, its application to complex clinical samples, particularly in secretome analysis, is constrained by several challenges, including labor-intensive sample preparation (e.g., protein enrichment and fractionation), interference from high-abundance proteins, limited dynamic range, and insufficient sensitivity and reproducibility for low-abundance targets (55, 56).In contrast, affinity-based proteomic platforms, particularly protein microarrays, offer distinct advantages in the detection of low-abundance proteins, with high sensitivity and specificity. These features make them especially well-suited for high-throughput analysis of complex clinical samples, positioning them as valuable complementary tools to MS-based approaches in both biomarker discovery and validation (57–60).

A protein microarray (or protein chip) is a high-throughput laboratory tool that immobilizes thousands of unique proteins on a solid surface, like a glass slide. It enables researchers to rapidly track specific antigen–antibody interactions and protein interactions, identify disease biomarkers, and screen for drug targets simultaneously using minimal sample volumes. This capability facilitates systematic profiling of protein expression patterns in complex biological samples and has established protein microarrays as indispensable tools in cancer proteomics research (47, 59, 61). Compared with conventional single-target assays such as enzyme-linked immunosorbent assay (ELISA), protein microarrays offer several key advantages, including high throughput, minimal sample consumption, and good reproducibility (59, 62). In addition, they enable the concurrent detection of multiple cytokines, chemokines, and growth factors, thereby facilitating the elucidation of the complex interactions among tumor cells, stromal components, and immune cells. Consequently, this technology has been widely applied in tumor biomarker discovery, signaling pathway analysis, as well as studies of the tumor microenvironment and its associated signaling networks (43, 46, 63–65).

In recent years, protein microarray-based proteomic platforms have been widely applied in gastric cancer (GC) and other malignancies, particularly for protein expression profiling in serum, tissue, and cellular samples, thereby facilitating the development and validation of multiplex protein biomarker panels. Notably, substantial progress has been achieved in GC research, where protein microarrays have been extensively utilized for biomarker discovery, signaling pathway analysis, molecular subtyping, and prognostic evaluation, demonstrating significant potential for clinical translation (30, 31, 66). Despite these advances, a comprehensive and systematic synthesis of protein microarray applications in GC remains lacking, particularly with respect to biomarker discovery, mechanistic investigation, and clinical translation. Therefore, a structured overview of recent developments in protein microarray technology is warranted to clarify its role within tumor proteomics and to highlight its potential in precision medicine. In this review, we summarize the fundamental principles and technical characteristics of protein microarrays, with a particular emphasis on their applications in biomarker identification, molecular mechanism exploration, and clinical diagnosis and prognostic assessment in GC. We also discuss current challenges and future perspectives to facilitate their translation into clinical practice.

## 2 Protein microarray technologies

In life science research and clinical diagnostics, proteins serve as the direct executors of biological functions, and their expression levels, post-translational modifications (PTMs), and interactions are critical for elucidating physiological and pathological mechanisms. However, conventional protein detection methods are typically limited by low throughput and an inability to simultaneously analyze multiple targets, making them insufficient for comprehensive analysis of complex biological samples.

The emergence of protein microarray technology has effectively addressed these limitations (62). Derived from DNA microarray principles (43), this approach involves immobilizing specific molecules onto solid supports (e.g., glass slides or nitrocellulose membranes) to construct miniaturized detection platforms. These platforms enable rapid, high-throughput, and parallel analysis of thousands of proteins and their modified forms in biological samples such as serum, plasma, and cell lysates (57, 58). Compared with gene expression profiling, protein microarrays directly interrogate the proteomic level, providing a more accurate reflection of cellular functional states and enabling detection of PTMs that cannot be inferred from RNA-level data (44, 67). Consequently, they have become essential tools in both basic research and translational applications (47, 68).

**Figure 1:**
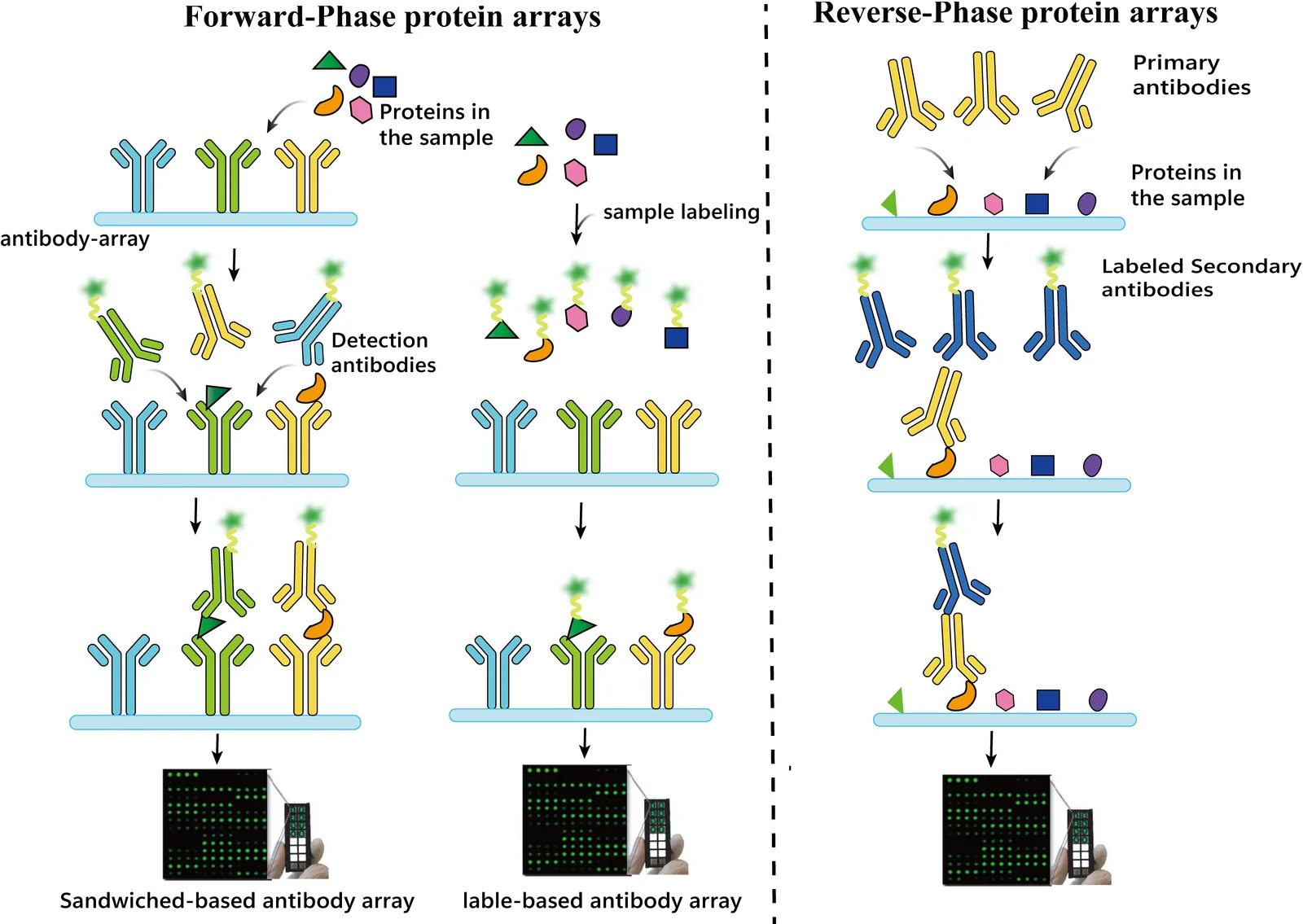
Schematic comparison of forward-phase and reverse-phase protein microarray platforms. The two left panels show the sandwich and label-based (direct) forward-phase assays, respectively. In the sandwich format, immobilized capture antibodies bind target proteins (circles, triangles, squares), and fluorophore- or enzyme-conjugated detection antibodies generate quantifiable signals. In the label-based format, pre-labeled protein samples (yellow stars) bind directly to capture antibodies, eliminating the need for a secondary detection step. The right panel depicts the reverse-phase protein array (RPPA), where complex protein lysates, serum, purified samples, or other biological samples are spotted onto the substrate, and the entire array is probed with a target-specific primary antibody, followed by fluorescent secondary antibody detection. Unlike forward-phase arrays that immobilize capture reagents to detect proteins in solution, RPPA immobilizes the samples themselves, enabling high-throughput screening of many samples against a single analyte. Different geometric shapes represent distinct proteins; yellow stars indicate fluorescent or enzymatic labels.

**Figure 2:**
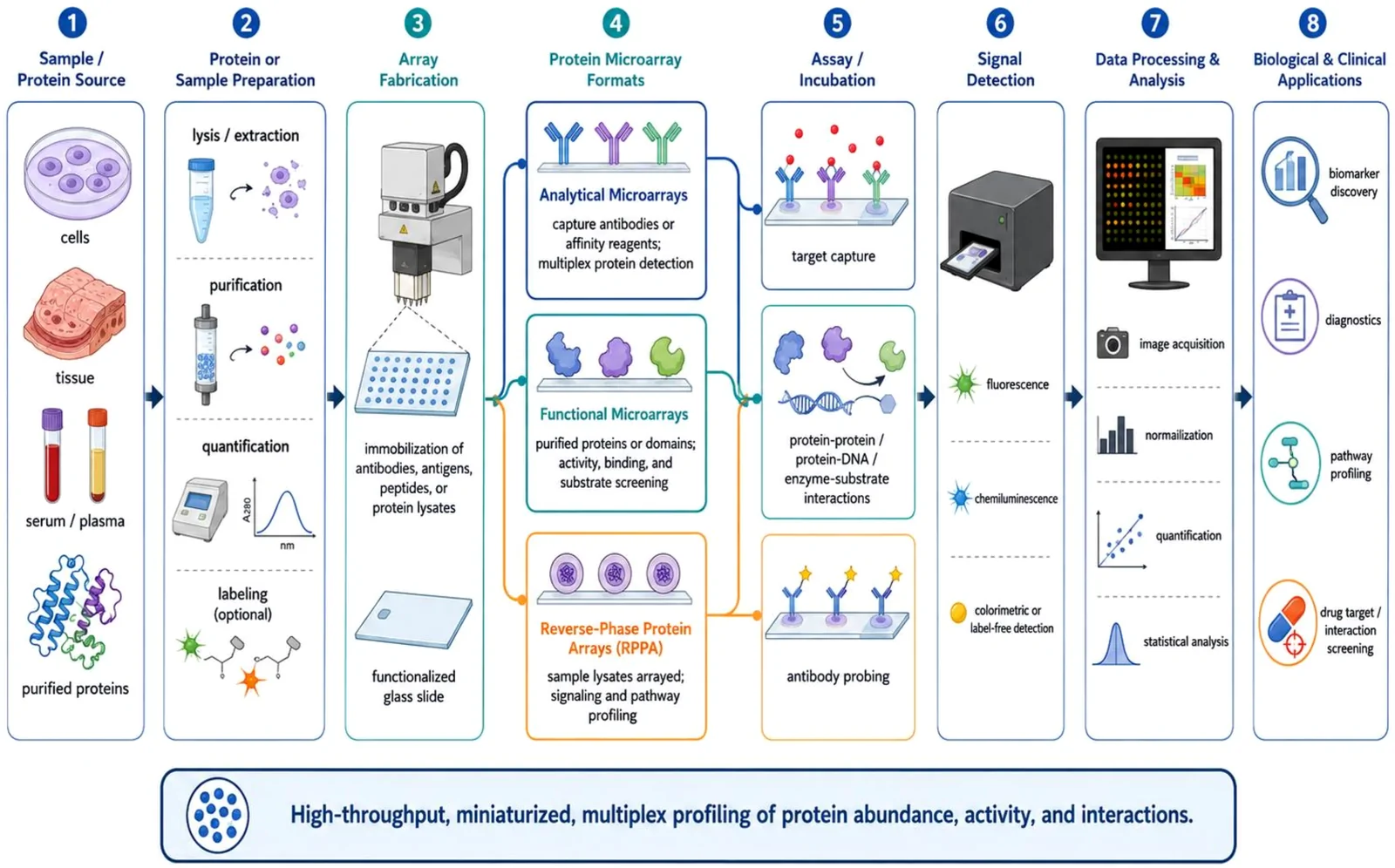
Workflow of protein microarray technologies. Protein microarray analysis begins with biological sample or protein source selection, followed by sample preparation, protein extraction, purification, quantification, and optional labeling. Functionalized glass slides or other solid supports are then fabricated by immobilizing capture antibodies, antigens, peptides, purified proteins, or sample lysates. Major platform formats include forward-phase analytical antibody microarrays for multiplex protein detection, functional protein microarrays for interaction or activity screening, and reverse-phase protein arrays (RPPAs) for sample-centric profiling of protein expression and signaling pathway activity. After assay incubation and signal detection by fluorescence, chemiluminescence, colorimetric, or label-free methods, array images are processed through normalization, quantification, and statistical analysis. These technologies enable high-throughput, miniaturized, multiplex profiling for biomarker discovery, molecular subtyping, pathway analysis, diagnostic research, therapeutic target identification, and treatment-response monitoring.

As a core technology in affinity-based proteomics, protein microarrays can be broadly categorized into two major platforms based on the immobilized component: forward-phase protein arrays (FPPA) and reverse-phase protein arrays (RPPA) (**Figures 1**, **2**) (67, 69).

### 2.1 Forward-phase protein arrays

FPPA immobilizes capture molecules—primarily highly specific antibodies or recombinant proteins—on a solid surface. These probes selectively bind target proteins in the sample, enabling simultaneous qualitative or quantitative detection of multiple analytes. Hundreds to thousands of proteins can be analyzed within a single sample (44, 57, 58). Based on detection strategies, FPPA is classified into two types:

#### 2.1.1 Sandwich-based antibody arrays

Sandwich-based antibody arrays employ a dual-antibody recognition strategy (capture antibody–target protein–detection antibody). This approach offers high specificity, sensitivity, and a broad dynamic range, but depends on well-matched antibody pairs, limiting scalability (57, 59, 70).

#### 2.1.2 Label-based antibody arrays

Label-based antibody arrays use a single-antibody capture strategy, with sample proteins pre-labeled (e.g., with biotin or fluorescent dyes) prior to incubation. This simplifies the workflow and facilitates high-density arrays, but labeling efficiency variability may affect quantitative accuracy (71, 72).

### 2.2 Reverse-phase protein array

In RPPA, biological samples (tissue/cell lysates, serum, etc.) are immobilized on the solid support (73, 74). Target proteins are detected using specific primary antibodies followed by labeled secondary antibodies, analogous to a miniaturized, high-throughput Western blot (73). Quantitative analysis is achieved through serial dilution and dose–response curve fitting (73). RPPA is particularly suited for large-scale cohort studies due to its “sample-centric” design, enabling simultaneous analysis of hundreds to thousands of samples using minimal material (73, 75). However, its performance heavily depends on antibody specificity and quality, and lacks molecular-weight separation (73, 75). Moreover, RPPA has proven broadly applicable—from tissue and liquid biopsy profiling to phosphorylation signaling and PTM analysis—and serves as a powerful tool for biomarker discovery, therapeutic monitoring, and disease classification (74–80).

### 2.3 Advantages and limitations of protein microarrays

As key affinity-based platforms, protein microarrays offer medium- to high-throughput profiling from minimal sample input, with the capacity to interrogate signaling pathways and PTMs (81–84). Their antigen–antibody-based detection is compatible with conventional immunoassays, facilitating validation and translation. However, performance is highly dependent on antibody specificity and quality, and batch-to-batch variability may affect reproducibility. Additionally, as a targeted technology, detection is confined to predefined panels, limiting unbiased discovery (82, 85). Future improvements in antibody reagents, detection sensitivity, and integration with computational approaches will further enhance their clinical utility.

## 3 Advances in protein microarray technologies for gastric cancer research

### 3.1 Biomarker discovery and early diagnosis

**Figure 3:**
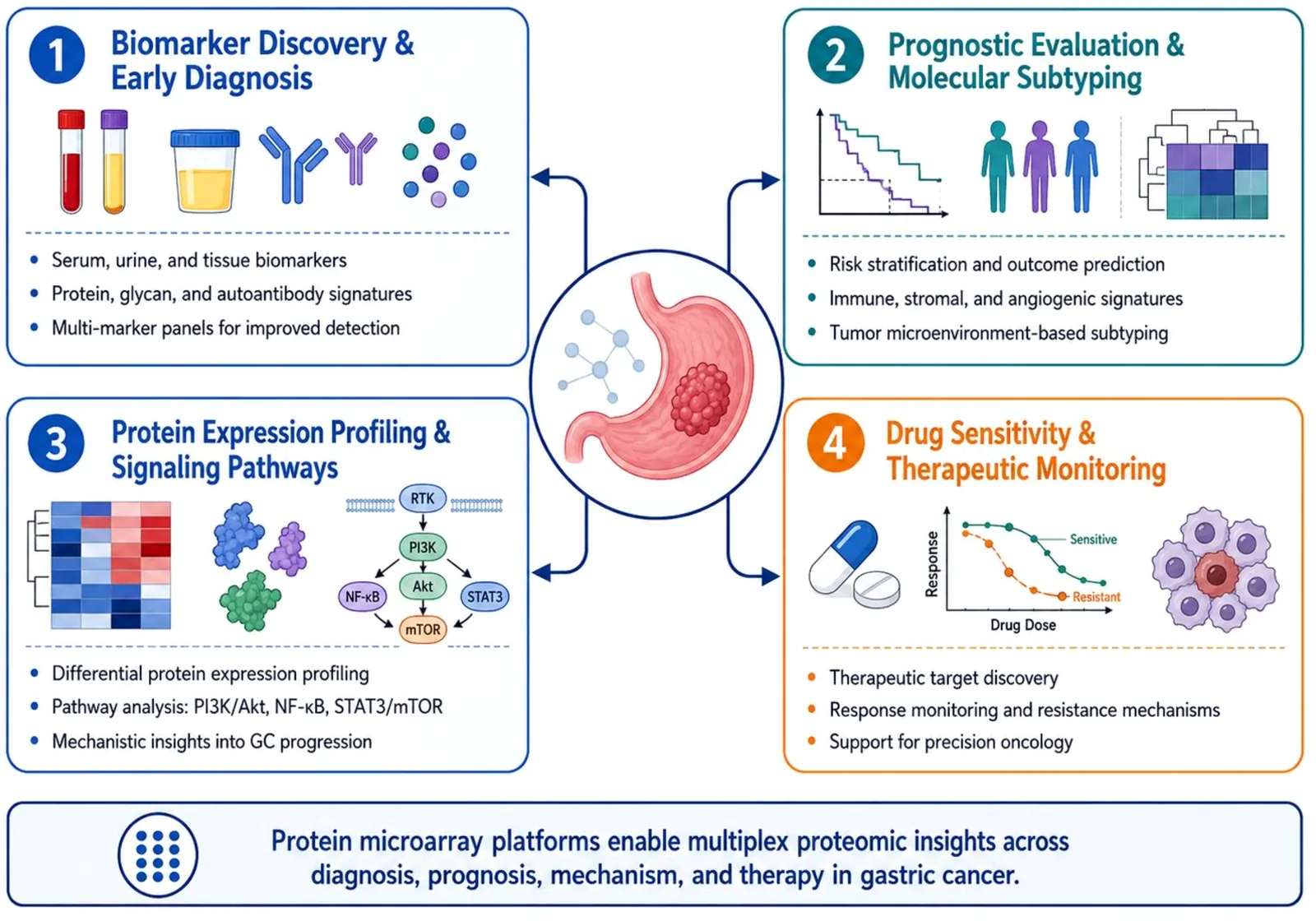
Applications of protein microarray technologies in gastric cancer research. Protein microarray platforms are widely used in gastric cancer research for (1) biomarker discovery and early diagnosis through the identification of serum, urine, and tissue biomarkers, as well as protein, glycan, and autoantibody signatures; (2) prognostic evaluation and molecular subtyping by defining risk-associated, immune, stromal, and angiogenic proteomic signatures; (3) protein expression profiling and signaling pathway analysis to reveal differentially expressed proteins and dysregulated pathways, including PI3K/Akt, NF-κB, and STAT3/mTOR; and (4) drug sensitivity assessment and therapeutic monitoring to support therapeutic target discovery, treatment-response evaluation, resistance mechanism studies, and precision oncology. Collectively, these applications demonstrate the value of protein microarray technologies in advancing diagnosis, prognosis, mechanistic understanding, and therapy development in gastric cancer.

Gastric cancer (GC) is frequently asymptomatic in its early stages, and conventional serum biomarkers such as carcinoembryonic antigen (CEA) and carbohydrate antigen 19-9 (CA19-9) exhibit limited sensitivity and specificity. Consequently, the identification of reliable, noninvasive biomarkers for early detection remains a critical unmet need. In recent years, protein microarray technology has emerged as a powerful platform for high-throughput biomarker discovery, enabling the simultaneous profiling of hundreds to thousands of proteins (**Table 1**; **Figure 3**).

**Table 1:** Summary of findings in gastric cancer research using protein microarrays: biomarker discovery and early diagnosis.

First author/s, year	Sample cohort/cell type	Sample types	Array type	Array results	(Refs.)
Yi et al, 2023	Screening: serum samples from HC, EGC, and AGC patients (88 samples) Validation: serum samples from HC, EGC, AGC, HCC, CCR, BC, and ACG patients (333 samples)	Human Serum	Sandwich-based FPPA	Identified 28 differentially expressed serum proteins associated with gastric cancer, including LAG-3, TGFβ RIII, CPA2, DCN, and ANGPTL3, Five proteins (LAG-3, TGFβ RIII, CPA2, DCN, ANGPTL3) showed significant differential expression and diagnostic potential for GC.	(86)
Wu et al, 2019	Serum from gastric cancer patients (stage IA) and healthy controls	Human Serum	Sandwich-based FPPA	Eleven cytokines were significantly elevated in gastric cancer serum vs controls: IFNGR1, Notch-3, TNFRSF19L, GHR, SLAMF8, FR-beta, integrin alpha 5, galectin-8, EphA1, epiregulin, FGF-12 (P < 0.05).	(28)
Ahn et al., 2012	Serum from gastric adenocarcinoma patients (n=215) and healthy controls (n=171)	Human Serum	29-plex antibody-based bead array (Luminex)	Two diagnostic algorithms (RF and SVM) were developed using panels of 8–11 biomarkers (EGFR, proApoA1, ApoA1, TTR, RANTES, D-dimer, vitronectin, IL-6, α2-macroglobulin, CRP, PAI-1). These panels distinguished gastric adenocarcinoma from controls with >85% accuracy in both training/test and independent validation sets.	(87)
Tong et al., 2016	Serum from gastric adenocarcinoma patients (n=285) and healthy controls (n=238)	Human Serum	9-plex antibody-based bead array (Luminex)	Five biomarkers (serum pepsinogen I, pepsinogen II, ADAM8, VEGF, and IgG to H. pylori) were selected as classifiers.	(27)
Yang et al., 2016	Serum from gastric cancer patients (n=537), healthy controls (n=550), and other gastric diseases (n=314)	Human Serum	Reverse-phase protein array (RPPA)	Four autoantibody biomarkers (COPS2, CTSF, NT5E, TERF1) were identified. The panel distinguished gastric cancer from healthy controls with 95% sensitivity and 92% specificity. These biomarkers were also associated with overall survival.	(31)
Yang et al., 2021	Serum from gastric adenocarcinoma patients (n=431) and healthy controls (n=381)	Human Serum	Reverse-phase protein array (RPPA)	Ten candidate TAAs (GNA11, GNAS, FGFR2, PBRM1, TP53, PIK3CA, COPB1, SRSF2, ACVR1B, SETBP1) were identified.	(30)
Fu et al., 2025	Serum from gastric cancer patients (n=296), healthy controls (n=265), and gastritis patients (n=195)	Human Serum	Reverse-phase protein array (RPPA)	Identified 24 IgA-related and 17 IgG-related differentially expressed autoantibodies between GC and controls; 20 IgA and 23 IgG DEAs between GC and gastritis. The IgG/IgA ratio of MIP1β showed highest AUC (0.81) for atrophic gastritis vs GC; MMP7 ratio best for chronic gastritis vs GC.	(29)
Xia et al., 2026	Discovery cohort: 29 EBVaGC + 33 EBVnGC (62 serum samples).	Human Serum	EBV proteome microarray (72 viral proteins, antigen-based)	Identified 34 EBV antigens with differential reactivities between EBVaGC and EBVnGC, revealing an IgG-skewed humoral signature with IgG-up/IgA-down patterns. A five-marker panel (LF2_IgG, BBLF2_IgG, BLRF2_IgG, BPLF1-2_IgA, BGLF4_IgA) was established based on the array screening results.	(88)
Shimura et al., 2015	Urine from gastric cancer patients (n=35) and healthy controls (n=35)	Human Urine	Sandwich-based FPPA	Expression levels of MMP-9, IL-8, and PECAM-1 were increased in urine of gastric cancer patients compared to healthy controls.	(89)
Hong et al., 2011	Urine from gastric cancer patients (n=7) and healthy controls (n=7)	Human Urine	Sandwich-based FPPA	Of 112 proteins predicted by computational method to be urine-excretory, 92 were detected in at least one urine sample (true positive rate >80%). The array data supported the classifier’s performance.	(90)
Cui et al., 2011	Serum from gastric cancer patients (n=9 for western blot, pooled for MS and array) and healthy controls (n=5 for western blot, pooled for MS and array)	Serum	label-based FPPA	Combined with mass spectrometry, the antibody array identified 67 out of 136 computationally predicted serum markers. Among these, 24 proteins showed differential abundance in gastric cancer sera vs controls, including elevated EGFR, GRO (CXCL1), IL-1α, osteoprotegerin (TNFRSF11B) and decreased sFRP-4, MMP-3, LIF, PARC (CCL18).	(32)
Rajkumar et al., 2010	Gastric tissue lysates (4 AN, 9 PN, 9 tumors) and plasma (58 gastric cancer, 18 non-malignant controls)	tissue lysates and plasma	Sandwich-based FPPA	Tissue: EpCAM, IL8, CCL4/MIP-1β, CCL20/MIP-3α, TIMP1 were significantly elevated in tumors vs normal tissues. Plasma: IL8, CXCL9/MIG, CCL3/MIP-1α, CCL20/MIP-3α, PDGFR-B, TIMP1 were significantly higher in gastric cancer patients vs non-malignant controls. Post-surgical plasma levels of EpCAM, IGFBP3, IL8, CXCL10/IP10, CXCL9/MIG, CCL3/MIP-1α, CCL20/MIP-3α, SPP1/OPN, PDGFR-B uniformly dropped in all 8 patients tested.	(91)
Liu et al., 2020	Serum from gastric cancer patients (n=66) and non-neoplastic gastric disease controls (n=34)	Serum	Sandwich-based FPPA	Serum levels of MMP3, MMP8, MMP9, TIMP1, and TIMP2 were significantly higher in GC group (p<0.05).	(92)
Xu et al., 2020	Serum from esophageal squamous cell carcinoma (ESCC) patients (n=20) and normal controls (n=20)	Serum	Label-based FPPA &Sandwich-based	IGFBP-1 was identified as significantly elevated in ESCC sera in both arrays.	(93)

GC, gastric cancer; HC, healthy controls; EGC, early gastric cancer; AGC, advanced gastric cancer; HCC, hepatocellular carcinoma; CCR, colorectal cancer; BC, breast cancer; ACG, atrophic chronic gastritis; FPPA, Forward Phase Protein Array; LAG-3, lymphocyte activating gene 3; TGFβ RIII, transforming growth factor-β receptor III; CPA2, carboxypeptidase A2; DCN, decorin; ANGPTL3, angiopoietin-like protein 3; IFNGR1, interferon gamma receptor 1; Notch-3, neurogenic locus notch homolog protein 3; TNFRSF19L, tumor necrosis factor receptor superfamily member 19L; GHR, growth hormone receptor; SLAMF8, signaling lymphocyte activation molecule family 8; FR-beta, folate receptor beta; EphA1, erythropoietin-producing hepatocellular A1; FGF-12, fibroblast growth factor 12; EGFR, epidermal growth factor receptor; proApoA1, pro-apolipoprotein A1; ApoA1, apolipoprotein A1; TTR, transthyretin; RANTES, regulated upon activation, normally T-expressed and presumably secreted; IL-6, interleukin-6; CRP, C-reactive protein; PAI-1, plasminogen activator inhibitor-1;ADAM8, A Disintegrin And Metalloproteinase domain-containing protein 8; VEGF, vascular endothelial growth factor; COPS2, COP9 constitutive photomorphogenic homolog subunit 2; CTSF, cathepsin F; NT5E, ecto-5’-nucleotidase; TERF1, telomeric repeat binding factor; GNA11, G protein subunit alpha 11; GNAS, GNAS complex locus (guanine nucleotide-binding protein alpha subunit); FGFR2, fibroblast growth factor receptor 2; PBRM1, polybromo 1; TP53, tumor protein p53; PIK3CA, phosphatidylinositol-4, 5-bisphosphate 3-kinase catalytic subunit alpha; COPB1, coatomer protein complex subunit beta 1; SRSF2, serine/arginine-rich splicing factor 2; ACVR1B, activin A receptor type 1B; SETBP1, SET binding protein 1; DEAs, differentially expressed autoantibodies; MIP1β, macrophage inflammatory protein-1 beta; MMP7, matrix metalloproteinase 7; EBVaGC, Epstein-Barr virus-associated gastric cancer; EBVnGC, EBV-negative gastric cancer; LF2, EBV early lytic protein; BBLF2, EBV early lytic protein; BLRF2, EBV tegument protein; BPLF1, EBV large tegument protein with deubiquitinase activity; BGLF4, EBV serine/threonine protein kinase; IL 8, Interleukin 8; PECAM 1, Platelet endothelial cell adhesion molecule-1; sFRP-4, secreted frizzled-related protein 4; IGFBP-1, insulin-like growth factor binding protein-1; ESCC, oesophageal squamous cell carcinoma.

In terms of circulating protein biomarkers, several studies have employed antibody microarrays for large-scale screening. Yi et al. (86) profiled 640 proteins and identified five differentially expressed candidates (TGFβRIII, LAG-3, CPA2, Decorin, and ANGPTL3), with the combination of CPA2 and TGFβRIII demonstrating promising diagnostic performance (AUC = 0.801). Similarly, antibody microarray–based serum profiling identified eleven significantly upregulated cytokines in gastric cancer, including IFNGR1, Notch‐3, TNFRSF19L, GHR, SLAMF8, and FR‐beta, and ELISA validation further confirmed these findings. Several of these proteins were reported for the first time to be elevated in GC serum, suggesting their potential utility as novel biomarkers for early diagnosis and prognosis evaluation (28).

Importantly, combinatorial biomarker panels consistently outperform single markers. A 29-plex serum biomarker platform integrating proteomics-derived and previously cancer-associated proteins identified multi-protein diagnostic algorithms composed of biomarkers such as EGFR, transthyretin (TTR), RANTES, and vitronectin (VN). These combinatorial biomarker panels achieved high diagnostic accuracies in both training/test (>88%) and independent validation (>85%) cohorts, highlighting their potential as minimally invasive tools for early gastric adenocarcinoma detection and risk stratification, while also complementing clinical gastroscopic evaluation of symptomatic patients to enhance diagnostic accuracy (87). Tong et al. (27) further established a multiplex biomarker panel (VEGF, ADAM8, pepsinogen I/II, and Helicobacter pylori IgG) using a Luminex platform, significantly improving diagnostic performance and supporting the utility of combined serum biomarkers in noninvasive GC screening. Collectively, these findings underscore the strong translational potential of multi-marker panels.

Autoantibodies against tumor-associated antigens (TAAs) have also emerged as promising biomarkers due to their high stability and early immune response. Wang et al. (31) identified a four-autoantibody biomarker panel (COPS2, CTSF, NT5E, and TERF1) using protein microarray technology, which demonstrated high sensitivity (95%) and specificity (92%) in distinguishing patients with gastric cancer from healthy controls, while also showing potential prognostic value. Similarly, focused protein array-based profiling of cancer driver gene–derived tumor-associated antigens identified serum autoantibody-based diagnostic panels for gastric adenocarcinoma, including a five-autoantibody signature (anti-TP53, anti-COPB1, anti-GNAS, anti–serine/arginine-rich splicing factor 2, and anti-SMARCB1), which demonstrated robust discriminative performance with AUC values ranging from 0.88 to 0.93 across training and independent validation cohorts, further supporting the diagnostic utility of anti-TAA autoantibody signatures (30). In addition, Fu et al. (29) demonstrated that integrating IgG- and IgA-related autoantibody profiles significantly improved diagnostic discrimination between gastric cancer, gastritis, and healthy controls. Notably, specific markers such as the IgG/IgA ratio of macrophage inflammatory protein-1β (MIP1β) and matrix metalloproteinase-7 (MMP7) showed strong diagnostic performance in distinguishing atrophic or chronic gastritis from gastric cancer. These findings highlight autoantibody profiling as a robust and noninvasive diagnostic strategy. Beyond autoantibodies, viral antigen-specific serological profiling is valuable for biomarker discovery in virus-associated gastric cancer. Xia et al. developed an EBV proteome microarray covering 72 viral proteins and identified a five-serum-marker panel (LF2_IgG, BBLF2_IgG, BLRF2_IgG, BPLF1-2_IgA, and BGLF4_IgA) that distinguished EBVaGC from EBVnGC with high accuracy (AUC = 0.933) and effectively screened EBVaGC from non-malignant gastric conditions in a community cohort (AUC = 0.942), establishing a noninvasive serological framework for EBVaGC diagnosis and population-level screening (88).

Beyond serum-based biomarkers, urine has also been explored as an alternative noninvasive source. Urinary levels of matrix metalloproteinase-9/neutrophil gelatinase-associated lipocalin (MMP-9/NGAL) complex and ADAM12 were found to be significantly elevated in patients with gastric cancer compared with healthy controls, and their combined detection achieved a diagnostic performance with an AUC of 0.825, suggesting their potential as noninvasive urinary biomarkers for gastric cancer, including early-stage disease (89). In parallel, computational approaches have been integrated into biomarker discovery workflows. For instance, Hong et al. (90) employed machine learning to predict urinary excretory proteins and identified endothelial lipase (EL) as a candidate biomarker, demonstrating the value of combining in silico prediction with experimental validation.

At the integrative omics level, multi-dimensional analyses have further expanded biomarker discovery. This study integrated transcriptomic profiling of gastric cancer tissues with computational prediction of secreted proteins to identify diagnostic biomarkers. Analysis of 80 paired samples revealed numerous differentially expressed genes and 21 co-expression clusters, with derived gene signatures showing strong classification accuracy. The study further prioritized 136 predicted secreted proteins and validated them at the serum level. Antibody array analysis, together with mass spectrometry, enabled high-throughput validation, detecting 81 serum proteins and identifying 29 with differential abundance between patients and controls. Overall, this multi-omics strategy supports robust serum biomarker discovery in gastric cancer (32). Similarly, integrated gene–protein analyses using transcriptomic profiling and antibody array validation identified consistent dysregulation of multiple cytokines, chemokines, and related factors in gastric cancer, including IL-8 (IL8), CXCL9/MIG, CXCL10/IP10, CCL3/MIP-1α, CCL20/MIP-3α, and TIMP1, as well as PDGFRB. These alterations were concordantly observed in both tumor tissues and patient plasma, supporting their potential diagnostic and disease monitoring relevance (91).

Targeted analyses of specific protein families have also yielded complementary insights. Liu et al. (92) demonstrated, using MMP antibody arrays, that multiple matrix metalloproteinases (MMPs) and their inhibitors (TIMPs) are significantly elevated in GC serum; although individual markers showed limited performance, combined detection substantially improved diagnostic accuracy (AUC > 0.8). Likewise, serum IGFBP-1, identified through antibody array screening and validated in large multi-center cohorts, has been shown to exhibit strong diagnostic performance for early-stage upper gastrointestinal cancers, including esophageal squamous cell carcinoma, esophagogastric junction adenocarcinoma, and gastric cancer, with AUC values ranging from 0.864 to 0.936, and its levels significantly decrease following surgical tumor resection (93).

In summary, protein microarray technologies have significantly advanced biomarker discovery in gastric cancer by enabling multidimensional profiling of protein expression, and autoantibody signatures. The shift from single biomarkers to multi-marker panels, coupled with integrative omics strategies, has markedly improved diagnostic performance. Future efforts focusing on large-scale validation, standardization, and clinical implementation will be essential to translate these findings into routine screening applications.

The consistent finding that multi-protein panels outperform single markers such as CEA and CA19–9 supports the adoption of combinatorial biomarker signatures for early GC detection, with several panels (e.g., EGFR/TTR/RANTES/VN) already validated in independent cohorts.

### 3.2 Prognostic evaluation and molecular subtyping

Beyond early detection, protein microarray technologies have become indispensable tools for prognostic evaluation and molecular subtyping in gastric cancer (GC). Increasing evidence indicates that disease progression and clinical outcomes are not determined solely by tumor-intrinsic alterations but are also shaped by dynamic interactions within the tumor microenvironment (TME), including immune cells, stromal components, and circulating tumor cells (CTCs). High-throughput protein profiling platforms enable systematic characterization of these complex interactions, facilitating the identification of clinically relevant prognostic biomarkers (**Table 2**; **Figure 3**).

**Table 2:** Summary of findings in gastric cancer research using protein microarrays: prognostic evaluation and molecular subtyping.

First author/s, year	Sample cohort/cell type	Sample types	Array type	Array results	(Refs.)
Han et al., 2023	Tumor interstitial fluid from 8 pairs of human gastric cancer tissues and matched adjacent normal tissues	Human gastric tissue	Sandwich-based FPPA	IL 1β expression was significantly upregulated in gastric cancer tissues compared with adjacent normal tissues (fold change >2 in all 8 pairs).	(66)
Longo et al., 2025	THP-1 cell line (no patient cohort for array; patient tissues used only for immunofluorescence validation)	Conditioned media and cell lysates from THP-1 macrophages (CLIC2 knockout vs control)	Sandwich-based FPPA	Cytokine array: In CLIC2-knockout macrophages vs control: increased secretion of CCL8, decreased secretion of IL-1β, IL-6, and osteoprotegerin (OPG); Phosphorylation array: increased phosphorylation of Shp1 and absence of Stat3 phosphorylation.	(94)
Shen et al., 2015	120 gastric cancer patients who underwent radical gastrectomy, with matched adjacent chronic gastritis tissues; median follow-up 68.5 months. Normal fibroblasts from one patient with normal gastric morphology (bariatric surgery)	Conditioned media from primary gastric inflammation-associated fibroblasts (GIAFs) and gastric cancer-associated fibroblasts (GCAFs)	sandwich-based FPPA	67 soluble mediators were differentially expressed between GCAFs and GIAFs. Key validated differences: GCAFs secreted higher levels of IL-6, IL-8, TGF-β1, HGF, IGF-1, CCL2, CCL5, CCL16, CCL28, CXCL9, CXCL12, and angiogenin compared to GIAFs.	(95)
Yang et al., 2017	6 pairs of CAFs and NFs isolated from gastric cancer patients (for antibody array)	Conditioned medium from cancer-associated fibroblasts (CAFs)	sandwich-based FPPA	CCL5 was identified as a key cytokine whose secretion was significantly reduced in KLF5-downregulated CAFs compared to control CAFs. The antibody array result guided subsequent ELISA validation, confirming that KLF5 regulates CCL5 production in CAFs.	(96)
Chen et al., 2014	Gastric cancer cell lines SGC7901 and HGC-27 (with JWA overexpression or knockdown, respectively)	Cell conditioned medium	sandwich-based FPPA	TIMP2 expression was significantly upregulated in conditioned medium from JWA-overexpressing cells and downregulated in JWA-knockdown cells. No consistent changes were observed for the other 42 proteins. This indicates that JWA inhibits MMP-2 activity and gastric cancer angiogenesis by positively regulating TIMP2.	(97)
Jin et al., 2021	Primary human umbilical vein endothelial cells (HUVECs) with TAGLN2 overexpression vs. mock control	Cell lysates (or conditioned medium) from HUVECs	sandwich-based FPPA	Angiogenesis array: Upregulation of TNFα, IL-8, IL-1β, IL-6, IL-1α, IL-12p40, IL-12p70, TGFα, IGF-1, PDGF-BB, EGF, HGF, G-CSF, GRO, PECAM-1 (CD31), VEGFR2, Tie-2; downregulation of uPAR, RANTES, ENA-78, ANG-2, LIF. Adhesion molecule array: Upregulation of CEACAM-1, NrCAM, ALCAM, ICAM-3; downregulation of E-cadherin, P-selectin, VCAM-1.	(98)
Li et al., 2023	BGC-823 gastric cancer cells with MAPK4 knockdown vs. control (shRNA)	Conditioned medium (serum-free, 24 h)	sandwich-based FPPA	40 cytokines were differentially secreted (fold change >1.5). Among them, 14 were upregulated upon MAPK4 depletion, including MIF (macrophage migration inhibitory factor), which was selected for further validation.	(99)
Rossi et al., 2025	Co-culture supernatants from: (i) isolated 3D compartment (PBMCs on Alvetex scaffold), (ii) isolated 2D compartment, (iii) complete co-culture (both compartments), with or without proliferating CTCs; also healthy donor PBMC co-cultures	Conditioned medium (neat)	sandwich-based FPPA	ENA-78 was secreted exclusively in the isolated 3D compartment and in the complete co-culture. Nine cytokines (GRO, GROα, IL-6, I-309, MCP-1, MCP-2, MCP-3, MCP-4) were detected only in the complete co-culture. No additional cytokines distinguished the presence or absence of proliferating CTCs.	(100)

CLIC2, Chloride intracellular channel 2; GCAFs, gastric cancer-associated fibroblasts; GIAFs, inflammation-associated fibroblasts; TGF-β1, transforming growth factor-β1; HGF, hepatocyte growth factor; CAFs, cancer-associated fibroblasts; NFs, normal fibroblasts; CCL5, C-C chemokine ligand 5; KLF5, Kruppel-like Zinc-Finger Transcription Factor 5; JWA, adenosine diphosphate-ribosylating-like factor 6 interacting protein 5 (ARL6ip5); MMP-2, matrix metalloproteinase-2; TIMP2, TIMP metallopeptidase inhibitor 2; HUVECs, human umbilical vein endothelial cells; TAGLN2, Transgelin 2; MAPK4, mitogen-activated protein kinase 4; MIF, macrophage migration inhibitory factor.

From the perspective of tumor–immune interactions, inflammation-driven metabolic reprogramming has emerged as a critical mechanism underlying immune evasion and poor prognosis in GC. Han et al. (66) demonstrated that IL-1β–induced acetylation of nicotinamide nucleotide transhydrogenase (NNT) maintains iron–sulfur cluster homeostasis, thereby promoting immune escape and resistance to immunotherapy. This IL-1β–NNT axis represents a novel metabolic–immune regulatory pathway associated with adverse clinical outcomes. In parallel, chloride intracellular channel protein 2 (CLIC2) has been shown to be highly expressed in macrophages within genomically stable GC, where it promotes differentiation toward an immunosuppressive phenotype and contributes to tumor progression (94). These findings suggest that immune-related protein signatures may serve as both prognostic indicators and markers of molecular subtypes.

Tumor–stromal interactions also play a pivotal role in GC progression. Cancer-associated fibroblasts (CAFs), as key components of the TME, exert profound effects on tumor behavior. Proteomic profiling of gastric cancer-associated fibroblasts identified Caveolin-1 (Cav-1) as a key regulator of fibroblast activation, with loss of Cav-1 promoting the secretion of pro-inflammatory and tumor-enhancing factors. Moreover, altered Cav-1 expression in stromal fibroblasts was significantly associated with GC progression and poor patient prognosis, highlighting its potential as an independent prognostic biomarker (95). In addition, elevated Kruppel-like factor 5 (KLF5) expression in CAFs was closely associated with aggressive clinicopathological characteristics and poor survival in GC patients. Mechanistically, KLF5-mediated activation of the CCL5/CCR5 signaling axis promoted gastric cancer cell progression, further emphasizing the critical role of stromal signaling pathways in tumor progression and prognostic evaluation (96).

Angiogenesis-related proteins represent another key class of prognostic biomarkers. Reduced JWA expression was shown to promote gastric cancer angiogenesis through enhanced Sp1-mediated transcriptional activation of matrix metalloproteinase-2 (MMP-2). Moreover, the combined expression pattern of low JWA and high MMP-2 was strongly associated with poor prognosis, indicating that these molecules may serve as independent prognostic biomarkers and potential therapeutic targets in GC (97). In addition, elevated transgelin-2 (Tagln2) expression in tumor-derived endothelial cells was found to promote angiogenesis through activation of neuropilin-1 (NRP1)/VEGFR2 signaling and downstream MAPK pathways. High Tagln2 expression was also significantly associated with lymph node metastasis, distant metastasis, and aggressive clinical progression, further underscoring the critical role of angiogenic protein networks in determining GC aggressiveness and prognosis (98).

At the level of tumor-intrinsic signaling, dysregulation of key kinases and signaling pathways further contributes to disease progression. Reduced expression of mitogen-activated protein kinase 4 (MAPK4) was significantly associated with liver metastasis and poor prognosis in gastric cancer patients. Mechanistically, MAPK4 silencing promoted a positive feedback loop between tumor cells and tumor-associated macrophages through macrophage migration inhibitory factor (MIF)-mediated signaling, thereby enhancing epithelial–mesenchymal transition and metastatic potential. These findings suggest that MAPK4 dysregulation may define a molecular subtype characterized by intensified tumor–immune crosstalk and aggressive metastatic behavior (99).

In addition, circulating tumor cells (CTCs) are key drivers of gastroesophageal cancer dissemination and are shaped by interactions with the peripheral immune system. Patient-derived CTC models from autologous immune co-culture show that 9p21.3 (CDKN2A/B and type I IFN cluster) deletion and 8q24.21 (MYC) amplification correlate with increased proliferation and immune evasion, reflecting tumor–immune selection pressure. CTC survival is not solely cell-intrinsic but supported by immune-derived signals. Antibody array–based proteomics identified a core cytokine network including MCP-1/CCL2, IL-6, and CXCL5, along with EV-associated signaling components, indicating a pro-inflammatory, tumor-promoting microenvironment. EV profiling further confirmed active CTC–immune communication and supports EV-mediated signaling in immune escape and metastasis. Together, antibody array–based proteomic and EV signatures define key protein targets governing CTC persistence and metastatic potential, providing insight into immune-driven regulation and its relevance for molecular subtyping and prognosis in gastroesophageal cancer (100).

Collectively, protein biomarkers identified through antibody arrays and other high-throughput proteomic platforms not only reflect tumor-intrinsic molecular alterations but also capture the complex interplay among cellular components within the TME. Integrating protein expression signatures with key functional pathways—including immune regulation, stromal remodeling, angiogenesis, and metastasis—offers a comprehensive framework for prognostic evaluation and molecular classification in gastric cancer. Future studies incorporating multi-omics integration and large-scale clinical validation will be essential to refine these biomarkers and accelerate their translation into precision oncology.

Protein signatures involving immune-related proteins (IL-1β, CLIC2), stromal factors (Caveolin-1, CCL5), and angiogenic mediators (MMP-2, Tagln2) have been independently associated with patient outcomes, offering potential for refining molecular subtyping and prognostic stratification.

### 3.3 Protein expression profiling and signaling pathway investigation in gastric cancer

The advances in antibody array–based proteomic technologies have substantially expanded the understanding of gastric cancer (GC) biology, particularly regarding tumor–stromal interactions, immune evasion, metastatic dissemination, cancer stemness, and therapy resistance. Compared to conventional single-target approaches, antibody arrays enable simultaneous profiling of multiple cytokines, chemokines, growth factors, and signaling mediators, thereby providing a systems-level view of the complex molecular networks underlying GC progression. Increasing evidence indicates that antibody array–driven analyses have uncovered several critical signaling axes involved in tumor microenvironment remodeling, angiogenesis, autophagy regulation, extracellular vesicle–mediated communication, and immune suppression (**Table 3**; **Figure 3**).

**Table 3:** Summary of findings in gastric cancer research using protein microarrays: protein expression profiling and signaling pathway investigation in gastric cancer.

First author/s, year	Sample cohort/cell type	Sample types	Array type	Array results	(Refs.)
Adithan et al., 2020	Extracellular compounds derived from SNU-484 gastric cancer cell culture supernatant (GC-EC)	Cell-conditioned medium concentrate (3 kDa cutoff, 10-fold concentrated)	sandwich-based FPPA	42 proteins were detected. The most abundant were MCP-1, angiogenin, Oncostatin M, IL-3, IL-8, M-CSF, VEGF-A, MCP-2, MCP-3, GRO-α, and others including IL-10, GM-CSF, RANTES, ENA-78, IFN-γ, TGF-β1, etc.	(101)
Zhu et al., 2016	Conditioned media from: (1) BMFs alone (BMF-CM), (2) BMFs + MKN28 co-culture (Co-culture-CM), (3) MKN28 alone (MKN28-CM)	Cell culture supernatants	sandwich-based FPPA	Mouse hemokine antibody array: Murine IL-6 was significantly higher in Co-culture-CM than in BMF-CM; TIMP-2 was also increased. Other detected factors included mTNF-α, mIL-1β, mIL-10, etc. Human cytokine antibody array: No major differences in human cytokines were reported in the text; the array was used to confirm that hIL-6, hTNF-α, hIL-1β levels remained low.	(102)
Zhao et al., 2023	Supernatants from HGC27 cells cultured alone or stimulated with PDPN(+) CAF-conditioned medium	Cell culture supernatants	Sandwich-based FPPA	IL-6 was identified as a key cytokine upregulated in CAF-stimulated GC cells compared to GC cells alone. Neutralizing IL-6 abrogated POSTN induction in PDPN(+) CAFs.	(103)
Sun et al., 2018	Conditioned media from GCMSCs (isolated from gastric cancer tissues of multiple patients) and BMMSCs (from healthy donors)	Cell culture supernatants	Sandwich-based FPPA	IL-6, IL-8, HGF, CCL11, MCP-2, MCP-3, and MIF were present at higher levels in GCMSC-CM compared to BMMSC-CM.	(104)
Zhao et al., 2025	Gastric cancer patients (tumor tissues)	Conditioned medium from PDPN(+) CAFs and PDPN(–) CAFs (sorted by FACS)	Sandwich-based FPPA	CCL2 was the most significantly overexpressed cytokine in PDPN(+) CAFs compared to PDPN(–) CAFs.	(105)
Cui et al., 2025	Conditioned media from WT and EFNA1 KO BGC-823 gastric cancer cells	Cell culture supernatants	Sandwich-based FPPA	CCL2 was the most significantly downregulated cytokine in EFNA1 KO cells compared to WT cells.	(106)
Yin et al., 2026	Conditioned media from CAFs isolated from gastric cancer tissues.	Cell culture supernatants (conditioned media)	Sandwich-based FPPA	Cytokine antibody array screening of CAF-conditioned media identified CCL5 as the most significantly secreted chemokine from CAFs.	(107)
Natsume et al., 2020	Gastric cancer cell lines (AGS, IM95) treated with conditioned media from omental adipocytes (OmAd-CM)	Cell culture supernatants (conditioned media)	Sandwich-based FPPA	GRO (CXCL2) was more abundant in OmAd-CM than in control media. No difference in GROα (CXCL1) levels was observed between OmAd-CM and control media.	(108)
Wang et al., 2020	Human peritoneal mesothelial cell line HMrSV5 exposed to normoxia or hypoxia (24 h)	Cell culture supernatants (conditioned media)	Sandwich-based FPPA	Hypoxic PMCs secreted significantly higher levels of several growth factors, including VEGFA, VEGFD, TGF-β2/3, and PDGF-AA/AB, compared to normoxic controls.	(109)
Zhao et al., 2026	Conditioned media from GCMSCs isolated from gastric cancer tissues	Cell culture supernatants	Sandwich-based FPPA	Cytokine array screening identified CCL11 as the most significantly upregulated chemokine secreted from GCMSCs.	(110)
Zhu et al., 2013	AGS gastric cancer cells with stable DARPP-32 overexpression vs. vector control	Cell lysates	Sandwich-based FPPA	No significant changes in the protein levels of the detected MMPs/TIMPs were observed between DARPP-32-overexpressing cells and control cells.	(111)
Wang et al., 2018	HSF-1 fibroblasts treated with exosomes from miR-27a-overexpressing gastric cancer cells vs. control exosomes	Cell culture supernatants	Sandwich-based FPPA	Several cytokines, including TNF-β, SDF-1, and FGF-4, were markedly differentially expressed between treatment and control groups.	(112)
Kalfon et al., 2022	Extracellular vesicles (EVs) isolated from AGS gastric cancer cell line	EV lysates (protein content)	Sandwich-based FPPA	High levels of proangiogenic proteins including ANG1, ANG2, VEGF, MMP1, uPAR, IL-6, and TIMP-1 were detected. Lower levels of antiangiogenic proteins such as angiostatin, endostatin, and IL-4 were also present.	(113)
Hsu et al., 2020	Conditioned media from M12 gastric cancer cells: control (pEGFP) vs. STK24 knockdown (sgSTK24-1.2)	Cell culture supernatants (serum-free, 48 h)	Sandwich-based FPPA	Secretion of IL-6, G-CSF, and CXCL16 was increased in STK24-knockdown cells compared to control.	(114)
Xu et al., 2018	Conditioned media from BGC823, BGC823/DDP, SGC7901, and SGC7901/DDP gastric cancer cells	Cell culture supernatants (serum-free, 24 h)	Sandwich-based FPPA	CCL2 was significantly upregulated in conditioned media of drug-resistant cells (BGC823/DDP and SGC7901/DDP) compared to their drug-sensitive parental cells.	(115)
Wang et al., 2021	Conditioned media from MKN-45 and MGC-803 gastric cancer cells with MALAT1 overexpression vs. control	Cell culture supernatants (serum-free)	Sandwich-based FPPA	IL-6 was significantly increased in the conditioned media of MALAT1-overexpressing cells compared to controls.	(116)
Liu et al., 2025	Gastric cancer stem cells (GCSCs) enriched from SGC-7901 and other GC cell lines, treated with clofoctol (CFT) vs. control	Cell lysates	Sandwich-based FPPA	CFT altered the expression of multiple apoptosis-related proteins, including increased cleaved caspase-3, cytochrome c, FasL, and decreased HSP27, Survivin, and XIAP.	(117)

MCP-1, Monocyte chemoattractant protein-1; M-CSF, Macrophage colony-stimulating factor; GM-CSF, Granulocyte-macrophage colony-stimulating factor; BMFs, bone marrow-derived myofibroblasts; HGF, hepatocyte growth factor; PDPN, podoplanin; CAF, cancer-associated fibroblast; POSTN, periostin; PDPN, podoplanin; CAFs, cancer-associated fibroblasts; FACS, fluorescence-activated cell sorting; CCL2, chemokine (CC-motif) ligand 2; EFNA1, ephrin A1; KO, knockout; VEGFA, vascular endothelial growth factor A; VEGFD, vascular endothelial growth factor D; TGF-β2/3, transforming growth factor-β2/β3; PDGF-AA/AB, platelet-derived growth factor-AA/AB; PMCs, peritoneal mesothelial cells; GCMSCs, gastric cancer-associated mesenchymal stem cells; CCL11, C-C motif chemokine ligand 11; MMP, matrix metalloproteinase; TIMP, tissue inhibitor of metalloproteinases; SDF-1, stromal cell-derived factor-1; ANG1, angiopoietin-1; ANG2, angiopoietin-2; VEGF, vascular endothelial growth factor; uPAR, not defined in the article; TIMP-1, tissue inhibitor of metalloproteinases-1; STK24, serine/threonine-protein kinase 24; G-CSF, granulocyte colony-stimulating factor; CXCL16, chemokine (C-X-C motif) ligand 16; DDP, cis-diamine dichlorodiplatinum (II) (cisplatin-resistant); MALAT1, metastasis-associated lung adenocarcinoma transcript 1.

One important application of antibody array in GC research has been the characterization of tumor-derived secretory factors that regulate immune escape and stromal remodeling. Adithan et al. demonstrated that gastric cancer cell-derived extracellular compounds (GC-ECs) selectively suppressed CD161^+^ CD3^−^ natural killer (NK) cells and promoted tumor formation in syngeneic mouse models (101). Cytokine array analysis identified multiple immunosuppressive cytokines and chemokines within GC-ECs, including MCP-1, IL-8, VEGF, angiogenin, and OSM, which collectively attenuated NK-cell cytotoxicity by reducing IFN-γ, granzyme B, and perforin expression. Activation of the PI3K/AKT signaling pathway further inhibited apoptosis and facilitated immune evasion, highlighting the utility of cytokine antibody arrays in identifying tumor-secreted immunomodulatory mediators involved in GC-associated immune suppression.

Antibody array–based profiling has also provided important insights into reciprocal signaling between gastric cancer cells and stromal fibroblastic components. Zhu et al. reported that bone marrow-derived myofibroblasts (BMFs) promoted gastric tumorigenesis and cancer stemness through a reciprocal IL-6/HGF/TGF-β1 signaling loop between stromal cells and GC cells (102). Co-culture analyses demonstrated that BMF-derived IL-6 and hepatocyte growth factor (HGF), together with tumor-derived TGF-β1, established a positive feedback circuit that activated JAK2/STAT3 signaling and enhanced stem cell-like phenotypes. Blockade of HGF/Met, JAK2/STAT3, or TGF-β1 signaling effectively suppressed sphere formation and tumor initiation, underscoring the importance of stromal–tumor communication in maintaining cancer stem cell properties.

Similarly, distinct subpopulations of cancer-associated fibroblasts (CAFs) exert profound regulatory effects on GC progression through cytokine-mediated signaling networks. In PDPN-positive CAFs, periostin (POSTN) was shown to enhance gastric cancer metastasis by regulating cancer stem cells through activation of the FAK/AKT and YAP signaling pathways (103). POSTN-induced FAK/AKT phosphorylation promoted stemness-associated phenotypes and metastatic potential, whereas activation of the FAK/YAP pathway stimulated IL-6 production in GC cells, thereby reinforcing PI3K/AKT signaling in PDPN(+)CAFs and sustaining POSTN secretion through a positive feedback loop. These observations emphasized the dynamic bidirectional communication between CAFs and GC stem-like cells during metastatic progression.

In addition to myeloid-driven immunosuppression, adaptive immune checkpoint regulation mediated by stromal components has emerged as a key complementary mechanism of immune evasion in gastric cancer. Recent studies have further expanded the immune suppression network by identifying mesenchymal stem cells (MSCs) as critical regulators of immune checkpoint activation in GC. Gastric cancer–derived mesenchymal stem cells (GCMSCs) were shown to induce programmed death-ligand 1 (PD-L1) expression in tumor cells through IL-8–mediated activation of STAT3/mTOR signaling and subsequent c-Myc–dependent transcriptional upregulation (104). Elevated PD-L1 expression markedly impaired CD8^+^T-cell cytotoxicity and reinforced an immune-excluded tumor microenvironment. Inhibition of IL-8 signaling effectively reduced PD-L1 expression and partially restored antitumor immune activity, indicating that the IL-8/STAT3/mTOR/c-Myc axis represents a critical stromal–tumor regulatory pathway controlling immune checkpoint activation. These findings position MSCs as upstream stromal regulators linking inflammatory cytokine signaling with adaptive T-cell exhaustion, thereby extending the immune suppression hierarchy beyond myeloid-mediated mechanisms observed in gastric cancer.

Beyond immune and stromal remodeling, antibody array technologies have substantially contributed to elucidating mechanisms underlying angiogenesis and metastatic niche formation in gastric cancer. Using integrated transcriptomic and functional analyses, PDPN(+) CAFs were identified as pro-angiogenic stromal subpopulation enriched in metastatic GC lesions (105). Activation of the AKT/NF-κB signaling pathway in PDPN(+) CAFs induced transcriptional upregulation of CCL2 through direct NF-κB p65 binding to the CCL2 promoter. Secreted CCL2 subsequently interacted with the atypical chemokine receptor ACKR1 on endothelial cells, thereby activating PI3K/AKT signaling and promoting endothelial migration and tube formation. Inhibition of either AKT signaling or the CCL2–ACKR1 axis effectively suppressed angiogenesis and tumor growth *in vivo*, highlighting the therapeutic relevance of stromal-derived chemokine signaling in GC.

Antibody array–based analyses have further revealed mechanisms governing organotropic metastasis. EFNA1-mediated signaling was identified as a critical regulator of hepatic premetastatic niche formation through induction of CCL2 secretion via the Hippo-YAP pathway (106). Elevated CCL2 activated hepatic stellate cells through WNT/β-catenin signaling, thereby facilitating liver metastasis. Pharmacological inhibition of CCL2 significantly reduced hepatic stellate cell activation and metastatic burden, suggesting that antibody array–based identification of secreted chemokines may facilitate development of anti-metastatic therapeutic strategies. Beyond chemokine-driven inflammatory reprogramming of the hepatic microenvironment, antibody array-based profiling has further uncovered metabolic signaling axes that couple lipid metabolism with metastatic dissemination. Using cytokine antibody array screening, Yin et al. identified CAF-derived CCL5 as a driver of gastric cancer metastasis through upregulation of GGT5, which mediates arachidonic acid metabolism reprogramming and EMT activation, ultimately promoting liver metastasis. This study illustrates how antibody array-based secretome profiling can uncover functionally relevant stromal-derived signaling axes (CCL5/GGT5) that couple lipid metabolism with metastatic progression in GC (107).

Peritoneal dissemination, one of the most clinically devastating manifestations of GC progression, has likewise been linked to cytokine-mediated remodeling within the peritoneal microenvironment. Omental adipocytes promoted GC peritoneal metastasis through secretion of CXCL2, which activated AKT/HIF1α signaling and induced VEGFA expression in gastric cancer cells (108). Cytokine profiling identified CXCL2 as a key adipocyte-derived mediator responsible for enhanced angiogenesis, migration, and tumor dissemination. Silencing CXCL2 abolished these pro-metastatic effects both *in vitro* and *in vivo*, while elevated urinary CXCL2 levels were associated with peritoneal metastasis in GC patients, indicating its potential utility as a biomarker for metastatic progression.

Complementary studies further demonstrated that hypoxia-driven stromal remodeling contributes substantially to peritoneal metastasis. Under hypoxic conditions, peritoneal mesothelial cells (PMCs) underwent autophagy-mediated degradation of SIRT1, resulting in increased HIF-1α acetylation and VEGFA secretion (109). Growth factor array screening identified VEGFA as a central mediator of PMC–tumor crosstalk. VEGFA subsequently activated VEGFR1 signaling in GC cells, triggering p-ERK/p-JNK activation and upregulation of integrin α5 and fibronectin expression, thereby enhancing cellular adhesion and metastatic colonization. Together, these findings established a mechanistic link between hypoxia, autophagy, and metastatic niche remodeling during GC peritoneal dissemination. Hypoxia-driven stromal remodeling is not limited to peritoneal dissemination but also extends to lymphatic metastasis, a key determinant of GC prognosis. By combining cytokine antibody array with transcriptomic and functional analyses, Zhao et al. established that hypoxia-inducible TCF21 transcriptionally upregulates CCL11 secretion from GCMSCs, which promotes gastric cancer lymph node metastasis through a paracrine mechanism involving CCR3-mediated M2 macrophage polarization and lymphatic endothelial cell remodeling, rather than through direct action on cancer cells. This study expands the hypoxia-centered metastatic paradigm beyond peritoneal and hematogenous routes, highlighting the critical role of GCMSC-derived chemokines in orchestrating stromal–immune–vascular crosstalk essential for lymphatic niche formation (110).

Antibody array–based analyses have also provided important insights into invasion-associated signaling pathways and extracellular vesicle–mediated intercellular communication. DARPP-32, which is overexpressed in a substantial proportion of gastric cancers, promoted GC cell invasion through stabilization of CXCR4 and activation of the MT1-MMP/MMP-2 signaling cascade (111). DARPP-32 directly interacted with CXCR4, prolonged its protein half-life, and reduced ligand-induced ubiquitination, thereby sustaining CXCR4-mediated signaling. Enhanced CXCR4 stability subsequently upregulated MT1-MMP expression and increased MMP-2 activity, ultimately facilitating extracellular matrix degradation and invasive behavior. Pharmacological inhibition or genetic silencing of CXCR4 significantly attenuated DARPP-32-mediated invasion, supporting the functional importance of the DARPP-32/CXCR4/MT1-MMP/MMP-2 axis in GC progression.

Increasing evidence also indicates that extracellular vesicle (EV)-mediated intercellular communication plays a crucial role in remodeling the tumor microenvironment. Exosomal miR-27a derived from GC cells induced the transformation of normal fibroblasts into cancer-associated fibroblasts through downregulation of CSRP2, thereby enhancing tumor proliferation, migration, and metastasis (112). These findings underscored the importance of exosome-mediated stromal reprogramming in GC progression.

In parallel, angiogenesis antibody array has been successfully employed to characterize the pro-angiogenic protein cargo of gastric cancer-derived EVs (113). GC-derived EVs were found to contain high levels of angiopoietin-2 (ANG2), which promoted endothelial cell proliferation, migration, invasion, and tube formation through activation of the PI3K/Akt signaling pathway. Silencing ANG2 effectively abolished the pro-angiogenic effects of GC-derived EVs, highlighting ANG2 as a critical mediator of EV-induced vascular remodeling.

Beyond tumor progression and metastatic dissemination, antibody array technologies have further facilitated mechanistic studies of therapy resistance, autophagy regulation, and therapeutic targeting in gastric cancer. Antibody array–guided analyses revealed that knockdown of serine/threonine-protein kinase 24 (STK24) promoted tumorigenesis and induced expansion of CD11b^+^ Ly6C^+^ myeloid-derived suppressor cells (MDSCs) and F4/80^+^ macrophages in orthotopic immunocompetent GC models (114). These observations suggested that dysregulation of kinase-mediated immune signaling contributes significantly to tumor progression and immune remodeling within the GC microenvironment.

One representative example of therapy-associated signaling adaptation is the identification of a CCL2–SQSTM1 positive feedback loop that promotes chemoresistance through suppression of pro-apoptotic autophagy (115). Cytokine screening revealed markedly elevated CCL2 secretion in chemoresistant GC cells compared to chemosensitive counterparts. CCL2 activated the PI3K-Akt-mTOR pathway, thereby suppressing autophagy and inducing accumulation of SQSTM1/p62. Increased SQSTM1 subsequently activated NF-κB signaling and further enhanced CCL2 transcription, establishing a self-sustaining feedback loop that maintained chemoresistance. Induction of autophagy or inhibition of CCL2 effectively restored chemosensitivity, suggesting that targeting cytokine-mediated autophagy suppression may represent a promising therapeutic strategy.

Autophagy-related signaling networks have also been implicated in GC progression through non-coding RNA regulation. LncRNA MALAT1 was reported to promote gastric cancer progression by inhibiting autophagic flux (116). Dysregulation of autophagy-associated pathways contributes to tumor survival, metastatic dissemination, and therapeutic resistance, further supporting the importance of autophagy-related protein signaling networks in GC biology.

Recent studies have further expanded the application of high-throughput signaling analyses toward therapeutic discovery targeting gastric cancer stem cells. Through large-scale drug screening approaches, clofoctol (CFT) was identified as an effective inhibitor of β-catenin signaling and GC stemness. Mechanistically, CFT directly bound the SUMO E3 ligase RanBP2, thereby suppressing the SerpinE1/β-catenin axis and activating TNF-mediated necroptosis through the RIPK1/RIPK3/MLKL pathway. CFT significantly reduced expression of stemness-associated markers, impaired tumor sphere formation, and suppressed tumorigenicity both *in vitro* and *in vivo*. These findings highlighted the translational potential of integrating proteomic signaling analyses with drug repurposing strategies for targeting GC stem cell populations (117).

Overall, these studies demonstrate that antibody arrays have become indispensable tools for deciphering the complex signaling networks underlying gastric cancer progression. By enabling high-throughput profiling of cytokines, chemokines, growth factors, extracellular vesicle cargo, and intracellular signaling mediators, antibody arrays have substantially advanced current understanding of tumor–microenvironment interactions, immune escape, angiogenesis, metastatic niche formation, autophagy regulation, cancer stemness, and therapy resistance. Importantly, these discoveries not only provide mechanistic insights into gastric carcinogenesis but also identify multiple potential biomarkers and therapeutic targets, thereby accelerating the development of precision medicine strategies for gastric cancer management.

Pathway-centric discoveries—such as the IL-8/STAT3/mTOR axis in PD-L1 upregulation and the CCL2–ACKR1 pro-angiogenic loop—not only elucidate GC biology but also nominate druggable targets for combination therapy.

### 3.4 Drug sensitivity and therapeutic monitoring

Recent advances in protein microarray–based proteomics have substantially improved the understanding of therapeutic response, chemoresistance, and tumor microenvironment–mediated drug adaptation in gastric cancer. By enabling high-throughput profiling of cytokines, receptor tyrosine kinases (RTKs), extracellular vesicle (EV)-associated proteins, and immune-related mediators, protein microarray technologies provide an efficient platform for identifying predictive biomarkers and uncovering molecular mechanisms underlying treatment sensitivity and resistance (**Table 4**; **Figure 3**).

**Table 4:** Summary of findings in gastric cancer research using protein microarrays: drug sensitivity and therapeutic monitoring.

First author/s, year	Sample cohort/cell type	Sample types	Array type	Array results	(Refs.)
Zhao ZX et al., 2023	Primary cancer-associated fibroblasts (CAFs) and normal fibroblasts (NFs) from gastric cancer patients; gastric cancer cell lines HGC-27 and AGS	Conditioned medium (CM) from CAFs and GC cells	Sandwich-based FPPA	IL-8 was the most significantly upregulated cytokine in CAF-CM compared to GC cell-CM (fold change 4.73). CAFs secreted higher levels of IL-8 than HGC-27 and AGS cells.	(118)
Kersy et al., 2019	Pooled exosomes from human omental tissue (n=4 patients) vs. pooled exosomes from subcutaneous fat (n=4 patients)	Exosome lysates	Sandwich-based FPPA	IL-6, IL-8, ICAM-1, GRO, bFGF, adiponectin, and CCL2 were increased in omental exosomes compared to subcutaneous fat exosomes.	(119)
Al-Marzouki et al., 2022	Malignant ascites from 7 stage IV gastric cancer patients; non-malignant cirrhotic ascites (n=1) as control	Cell-free ascites fluid	Sandwich-based FPPA	ANG-2, HGF, IL-8, TIMP-2, uPAR, VEGF, NAP-2, and MIF were elevated in malignant ascites vs. cirrhotic control; only VEGF and MIF passed multiple testing correction (Bonferroni adjusted p=0.00055 and p=0.0428, respectively).	(120)
Tang et al., 2018	SGC7901 gastric cancer cells treated with ADR alone vs. ADR + PMI	Cell lysates	Sandwich-based FPPA	Compared to ADR alone, the combination of ADR and PMI decreased the phosphorylation levels of EGFR, FAK, Tyk2, ROS, and LTK.	(121)
Zhu et al., 2011	MKN-28 gastric cancer cells stably expressing DARPP-32 or empty vector, treated with gefitinib (25 μM) overnight	Cell lysates	Sandwich-based FPPA	DARPP-32-expressing cells showed significantly higher levels of total EGFR (5-fold) and p-EGFR(Y845) (4-fold) compared to control cells after gefitinib treatment.	(122)
Yoon et al., 2012	SNU484 gastric cancer cells treated with DMSO or sunitinib (0.1 μmol/L; SNU484 cells transfected with control siRNA or PDGFRA siRNA	Conditioned media (cell culture supernatants)	Sandwich-based FPPA	Sunitinib treatment reduced the levels of several angiogenic cytokines including bFGF, IFN-γ, IGF-1, PDGF-BB, and VEGF compared to DMSO control. PDGFRA knockdown reduced angiogenic cytokine secretion similar to sunitinib treatment, indicating that sunitinib’s anti-angiogenic effects are mediated via PDGFRA inhibition.	(123)
Torti et al., 2011	GTL16 gastric cancer cells (MET-amplified, Met-addicted) treated with Met inhibitor PHA-665752 or DMSO	Conditioned Media (Cell culture supernatants)	Sandwich-based FPPA	Met inhibition led to decreased secretion of GROα, IL-8, IGFBP-1, IL-6R, uPAR, STNFR2, OPG, and increased secretion of MCP-1, IL-6, and biphasic changes in TIMP-2.	(124)
Miao et al., 2020	Supernatants from human PBMC-derived macrophages (PBMC-DMs) with STING knockdown, STING overexpression, STING activation (2′3′-c-GAMP), or control	Cell culture supernatants	Sandwich-based FPPA	L6R was identified as one of the most significantly changed cytokines in both STING knockdown and STING-activated macrophages. Pathway analysis of differentially expressed cytokines revealed enrichment of the JAK-STAT signaling pathway.	(125)
Wang et al., 2026	Gastric cancer cells (MGC803, HGC27) treated with oxaliplatin and 5-FU combination vs. control	Cell culture supernatants (conditioned media)	Sandwich-based FPPA	Levels of NK cell recruitment-related chemokines including CCL3, CCL5, CCL7, CCL8, CCL13, and CCL16 were significantly increased in the supernatant of chemotherapy-treated tumor cells compared to control.	(126)
Zhu et al., 2022	Co-culture supernatants from ZFP64-overexpressing HGC-27 gastric cancer cells vs. control cells co-cultured with PBMCs (peripheral blood mononuclear cells)	Cell culture supernatants	Sandwich-based FPPA	ZFP64 overexpression was associated with decreased levels of effector cytokines IFN-γ and IL-2, and increased levels of immunosuppressive cytokines including CXCL10, CXCL1, CCL3, DKK1, CD71, IL-1α, CSF1, and IL-6.	(127)
Kato et al., 2012	MKN74 gastric cancer cells (*in vitro*) and MKN74 xenograft tumor tissues (*in vivo*) with or without metformin treatment	Cell lysates and tissue lysates	Sandwich-based FPPA	Metformin treatment reduced the expression levels of p-EGFR and p-IGF-1R both *in vitro* and *in vivo* compared to untreated controls.	(128)
Shah et al., 2019	KATO-III gastric signet ring cell adenocarcinoma cells (adherent vs. non-adherent populations)	Cell culture supernatants (serum-free, 24 h)	Sandwich-based FPPA	Significant differences in cytokine secretion between adherent and non-adherent cells: adherent cells secreted higher levels of TIMP-4, Activin A, NGFR, IL-2Rγ, and IL-2IR; non-adherent cells secreted higher levels of SCFR, MMP-13, EGF, MMP-1, Prolactin, TGF-α, and Siglec-5 (fold change >5, P<0.05).	(129)
Hara et al., 2016	MKN-28 gastric cancer cells and cancer-associated fibroblasts (CAFs) isolated from human colon cancer	conditioned media (Cell culture supernatants)	Sandwich-based FPPA	MKN-28 cells secreted IL-8, TIMP-1, TIMP-2, CXCL13, and uPAR (no VEGF); itraconazole did not alter their secretion. CAFs secreted IL-1β, VEGF, TIMP-1, MCP-1, and PAI-1; itraconazole selectively suppressed MCP-1 secretion.	(130)
Zhou et al., 2016	BALB/c mice treated with BF-rTK or E. coli DH5α via intramuscular (IM) or intravenous (IV) injection at high (1×10^6^ cells/mL) or low (1×10^4^ cells/mL) doses	Mouse serum	Sandwich-based FPPA	IM injection of high-dose BF-rTK upregulated IL-5, IL-6, IL-9, IL-10, IL-13, IL-17, KC, GM-CSF, M-CSF (>10-fold) and IL-1α, IL-2, IL-12 (>2-fold), while reducing RANTES and VEGF. Low-dose IM BF-rTK upregulated IL-5, IL-6, IL-9, IL-10, KC, GM-CSF (>10-fold) and IL-1α, IL-1β, IL-2, IL-17, MCP-1 (>2-fold). IV injection of low-dose BF-rTK induced minimal cytokine changes (only IL-1α, IL-10, GM-CSF >10-fold; IL-2 >2-fold) and reduced IL-6, IFN-γ, RANTES.	(131)
Li et al., 2019	MFC tumor-bearing 615 mice treated with BSP (800 μg/g body weight, i.p.) or saline	Mouse serum	Sandwich-based FPPA	BSP treatment significantly increased serum levels of GM-CSF, IL-2, IL-6, IL-9, IL-10, IL-13, IFN-γ, MCP-1 (CCL2), SCF, and MCP-5 compared to saline controls.	(132)

CAFs, cancer-associated fibroblasts; NFs, normal fibroblasts; GC, gastric cancer; CM, conditioned medium; ICAM-1, intercellular adhesion molecule-1; GRO, growth related oncogene; bFGF, basic fibroblast growth factor; adiponectin, adiponectin; CCL2, C-C Motif Chemokine Ligand 2; MIF, macrophage migration inhibitory factor; EGFR, epidermal growth factor receptor; FAK, focal adhesion kinase; DARPP-32, Dopamine and cAMP-regulated phosphoprotein, Mr 32000; EGFR, epidermal growth factor receptor; IFN-γ, interferon-gamma; IGF-1, insulin-like growth factor 1; PDGF-BB, platelet-derived growth factor BB; VEGF, vascular endothelial growth factor; PDGFRA, platelet-derived growth factor receptor alpha; GROα, growth-related oncogene α; IGFBP-1, insulin-like growth factor binding protein 1; IL-6R, interleukin-6 receptor; uPAR, plasminogen activator urokinase receptor (also known as PLAUR); STNFR2, tumor necrosis factor receptor superfamily member 1B; OPG, tumor necrosis factor receptor superfamily member 11B (also known as TNFSR11B); MCP-1, chemokine (C-C motif) ligand 2 (also known as CCL2); TIMP-2, TIMP metallopeptidase inhibitor 2; STING, stimulator of interferon genes; PBMC, peripheral blood mononuclear cells; ZFP64, Zinc Finger Protein 64; PBMCs, peripheral blood mononuclear cells; TIMP-4, tissue inhibitor of metalloproteinase-4; NGFR, nerve growth factor receptor; SCFR, stem cell factor receptor; MMP-13, matrix metalloproteinase-13; EGF, epidermal growth factor; MMP-1, matrix metalloproteinase-1; Prolactin, prolactin; TGF-α, transforming growth factor-α; Siglec-5, sialic acid binding immunoglobulin-like lectin-5.

Several studies have demonstrated that protein microarray–based profiling can identify key signaling pathways associated with chemotherapy resistance. Cancer-associated fibroblasts (CAFs) were shown to promote oxaliplatin resistance through IL-8–mediated activation of the PI3K/AKT pathway, whereas calcipotriol effectively reversed this effect by blocking PI3K/AKT signaling (118). Using cytokine antibody arrays combined with transcriptomic analyses, IL-8 was identified as a key CAF-derived mediator of chemoresistance. Studies have shown that omentum-derived conditioned medium and exosomes significantly enhance the proliferation, migration, and invasion of gastric cancer cells, while also increasing resistance to platinum-based chemotherapy. Proteomic analyses have identified IL-6, IL-8, MMP9, FN1, and CXCL5 as key mediators of these pro-metastatic and chemoresistant effects (119). Similarly, malignant ascites promotes the adhesion of gastric cancer cells to peritoneal mesothelial and peritoneal tissues, with sustained overexpression of MIF and VEGF identified as central drivers of peritoneal metastasis; functional blockade of these factors effectively suppresses tumor implantation and dissemination within the peritoneal cavity (120). Collectively, the omental microenvironment and malignant ascites constitute critical ecological niches that facilitate gastric cancer peritoneal metastasis. They act through a coordinated network of soluble factors, including IL-6, IL-8, CXCL5, VEGF, ICAM-1, and CCL2, to regulate tumor cell adhesion, invasion, and chemoresistance. These findings highlight the value of antibody array- and proteomics-based secretome profiling in systematically elucidating the mechanisms of gastric cancer peritoneal dissemination and in identifying potential therapeutic targets.

Antibody phosphorylation arrays have also been widely applied to identify drug-responsive signaling pathways and therapeutic sensitizers. Peiminine was shown to enhance adriamycin sensitivity in gastric cancer through suppression of the EGFR/FAK signaling pathway, as demonstrated using RTK phosphorylation antibody arrays (121). In addition, DARPP-32 promoted gefitinib resistance by stabilizing EGFR and enhancing EGFR/ERBB3 interactions, leading to sustained PI3K/AKT activation and resistance to EGFR-targeted therapy (122). Sunitinib further exhibited synergistic antitumor effects when combined with cisplatin by promoting PDGFRA-dependent downregulation of ERCC1 and suppressing DNA repair signaling, thereby reducing DNA repair capacity and enhancing cisplatin sensitivity (123). Collectively, these studies demonstrate the utility of antibody-based phosphoproteomics for identifying actionable signaling networks involved in targeted therapy resistance and chemosensitization.

In addition to identifying resistance mechanisms, antibody arrays have facilitated the discovery of predictive biomarkers for therapeutic monitoring. Torti et al. established a preclinical biomarker algorithm for MET-targeted therapy in MET-amplified gastric cancer using antibody proteomics and gene expression profiling. The study identified soluble IL-8, GROα, uPAR, and IL-6 as surrogate biomarkers dynamically associated with pharmacological MET inhibition, suggesting their potential utility for monitoring treatment response during targeted therapy (124).

Emerging evidences also indicate that protein microarray technologies are valuable for evaluating immunotherapy-associated remodeling of the gastric cancer microenvironment. Targeting the STING pathway in tumor-associated macrophages (TAMs) altered inflammatory cytokine secretion profiles and enhanced innate immune sensing of gastric cancer cells (125). Neoadjuvant chemotherapy was further shown to induce CCL8-dependent recruitment of natural killer (NK) cells, thereby enhancing antitumor immune activity and improving therapeutic efficacy (126). Moreover, targeting the ZFP64/GAL-1 axis significantly enhanced the efficacy of nab-paclitaxel while reversing the immunosuppressive microenvironment in gastric cancer (127). These studies collectively emphasize the importance of immune-associated proteomic signatures in therapeutic monitoring and immunomodulatory treatment strategies.

Beyond resistance-associated signaling, antibody array–related approaches have also contributed to the identification of alternative therapeutic strategies. Metformin inhibited gastric cancer proliferation both *in vitro* and *in vivo* through suppression of proliferative signaling pathways and alterations in growth-associated protein expression (128). Differentiation-targeting strategies in KATO III gastric cancer cells reduced epithelial–mesenchymal transition (EMT) and cancer stem cell markers, suggesting potential applications for overcoming metastasis and therapeutic resistance (129). Furthermore, itraconazole suppressed CAFs and endothelial cells in bevacizumab-resistant gastrointestinal cancer, highlighting the therapeutic potential of targeting stromal components within the tumor microenvironment (130). Additional experimental therapeutic approaches, including Bifidobacterium-mediated HSV-1 thymidine kinase/ganciclovir gene therapy and anti-tumor polysaccharide treatment, also demonstrated significant antitumor activity in gastric cancer models (131, 132). Studies have demonstrated that the Bifidobacterium-mediated herpes simplex virus thymidine kinase/ganciclovir (HSV-TK/GCV) suicide gene therapy significantly inhibits tumor growth in gastric cancer models, while intravenous administration maintains antitumor efficacy without inducing evident systemic toxicity or immune-related adverse effects (131). Similarly, the natural bamboo shaving polysaccharide (BSP) exhibits potent antitumor activity in gastric cancer models by promoting tumor cell apoptosis and enhancing antitumor immune responses, including activation of T cells and natural killer (NK) cells, thereby remodeling the tumor microenvironment and prolonging survival (132). Overall, these experimental therapeutic strategies highlight distinct yet complementary mechanisms—gene therapy- and immunomodulation-based approaches—that demonstrate robust antitumor efficacy and promising translational potential in gastric cancer.

Collectively, these studies demonstrate that protein microarray technologies provide a powerful platform for decoding therapeutic response mechanisms, identifying resistance-associated signaling pathways, and discovering predictive biomarkers in gastric cancer. Integration of cytokine arrays, phosphoproteomics, EV profiling, and immune-associated protein signatures is expected to further advance precision medicine and individualized therapeutic monitoring in gastric cancer.

Identification of resistance-related proteins (e.g., IL-8, CCL2, DARPP-32) and surrogate biomarkers (IL-8, GROα, uPAR) provides a rationale for patient selection, treatment monitoring, and early detection of resistance, paving the way for precision oncology in GC.

### 3.5 Key proteins identified by protein microarray in gastric cancer

Through the studies summarized above, a non-redundant set of proteins has been consistently identified and validated across multiple cohorts. These proteins can be categorized by their primary clinical utility:

#### 3.5.1 Diagnostic biomarkers (early detection)

Through the studies summarized above, a non-redundant set of proteins has been consistently identified and validated across multiple cohorts. **Table 5** summarizes these key proteins categorized by their primary clinical utility.

**Table 5:** Non-redundant key proteins identified by protein microarray in gastric cancer.

Clinical application category	Protein(s)/panel	Key finding/application	Reference(s)
Diagnostic biomarkers (early detection)	EGFR, TTR, RANTES, VN (multi-protein panel)	Distinguishes GC from controls, AUC > 0.85	(87)
	COPS2, CTSF, NT5E, TERF1 (autoantibody panel)	95% sensitivity, 92% specificity	(31)
	TGFβRIII, CPA2, LAG-3 (serum proteins)	Showed promising diagnostic performance	(86)
Prognostic biomarkers (risk stratification & subtyping)	IL-1β/NNT acetylation axis	Correlates with immune evasion and poor survival	(66)
	CLIC2 (macrophage expression)	Defines an immunosuppressive subtype in genomically stable GC	(94)
	Caveolin-1 (loss in stromal fibroblasts)	Associated with aggressive progression and poor prognosis	(95)
	CCL5/CCR5 (CAF-derived signaling axis)	Promotes metastasis and worse outcomes	(96)
Therapeutic response & resistance markers	IL-8 (CAF-derived)	Mediates oxaliplatin resistance via PI3K/AKT	(118)
	CCL2–SQSTM1 positive feedback loop	Suppresses autophagy, promotes chemoresistance	(115)
	DARPP-32	Stabilizes EGFR, confers gefitinib resistance	(122)
	Soluble IL-8, GROα, uPAR	Proposed as surrogate biomarkers for monitoring MET-targeted therapy	(124)

This table summarizes non-redundant proteins that have been consistently validated across multiple cohorts using protein microarray technologies in gastric cancer. Proteins are grouped according to their primary clinical utility. AUC, area under the curve; CAF, cancer-associated fibroblast; EGFR, epidermal growth factor receptor; TTR, transthyretin; RANTES, regulated upon activation, normal T-cell expressed and presumably secreted; VN, vitronectin; COPS2, COP9 signalosome subunit 2; CTSF, cathepsin F; NT5E, ecto-5′-nucleotidase; TERF1, telomeric repeat-binding factor 1; TGFβRIII, transforming growth factor-β receptor III; CPA2, carboxypeptidase A2; LAG-3, lymphocyte activation gene 3; NNT, nicotinamide nucleotide transhydrogenase; CLIC2, chloride intracellular channel protein 2; CCL5, C-C motif chemokine ligand 5; CCR5, C-C chemokine receptor type 5; CCL2, C-C motif chemokine ligand 2; SQSTM1, sequestosome-1; DARPP-32, dopamine- and cAMP-regulated phosphoprotein, Mr 32000; GROα, growth-regulated oncogene α; uPAR, urokinase-type plasminogen activator receptor.

Multi-protein panels (EGFR, TTR, RANTES, VN) achieved AUC >0.85 in distinguishing GC from controls (87); autoantibody panels (COPS2, CTSF, NT5E, TERF1) reached 95% sensitivity and 92% specificity (31); serum proteins including TGFβRIII, CPA2, and LAG-3 also showed promising diagnostic performance (86).

#### 3.5.2 Prognostic biomarkers (risk stratification and subtyping)

Elevated IL-1β and its downstream NNT acetylation axis correlate with immune evasion and poor survival (66); CLIC2 expression in macrophages defines an immunosuppressive subtype in genomically stable GC (94); loss of Caveolin-1 in stromal fibroblasts is associated with aggressive progression (95); CCL5/CCR5 signaling from CAFs promotes metastasis and worse outcomes (96).

#### 3.5.3 Therapeutic response and resistance markers

IL-8 mediates CAF-driven oxaliplatin resistance via PI3K/AKT (118); CCL2-SQSTM1 feedback loop suppresses autophagy and promotes chemoresistance (115); DARPP-32 stabilizes EGFR to confer gefitinib resistance (122); soluble IL-8, GROα, and uPAR have been proposed as surrogate biomarkers for monitoring MET-targeted therapy (124).

## 4 Discussion

### 4.1 Key findings and contextualization within prior research

In this review, we systematically summarize the application of protein microarray–based proteomic technologies in gastric cancer (GC) research, with a particular focus on biomarker discovery, molecular subtyping, signaling pathway investigation, and therapeutic monitoring. The key findings emerging from the literature can be summarized as follows.

First, protein microarrays have enabled a paradigm shift from single-marker to multi-protein signature–based diagnostics in GC. Studies consistently demonstrate that panels comprising multiple cytokines, autoantibodies, or matrix metalloproteinases (MMPs) significantly outperform conventional single markers such as CEA and CA19-9 (27–29, 86, 87). This finding aligns with the broader trend in cancer biomarker research, where the inherent heterogeneity of malignancies necessitates combinatorial approaches. Notably, the integration of autoantibody signatures (e.g., anti-TP53, anti-COPB1) has extended the diagnostic window by capturing early host immune responses, which is particularly valuable for early-stage GC where circulating protein levels may remain within normal ranges (30, 31).

Second, protein microarrays have substantially advanced our understanding of tumor microenvironment (TME) dynamics in GC. Beyond merely cataloging differentially expressed proteins, antibody array–based studies have unraveled complex paracrine signaling loops—such as the IL-6/HGF/TGF-β1 feedback circuit between bone marrow-derived myofibroblasts and GC cells (102), and the CCL2–ACKR1 axis in PDPN(+) cancer-associated fibroblasts (105)—that drive cancer stemness, angiogenesis, and metastasis. These findings complement and extend earlier genomic studies (39, 40) by providing functional, protein-level evidence of how stromal components actively shape tumor behavior.

Third, the application of protein microarrays to therapy response assessment has identified multiple resistance-associated pathways, including IL-8–mediated PI3K/AKT activation in CAF-driven oxaliplatin resistance (118) and DARPP-32–dependent EGFR stabilization in gefitinib resistance (122). Importantly, these studies have also nominated actionable targets, such as CCL2 in chemoresistance (115) and PDGFRA in cisplatin sensitivity (123), thereby directly informing combination therapy strategies.

### 4.2 Potential shortcomings and limitations

Despite the promising findings summarized in this review, several limitations must be acknowledged when interpreting the existing literature and when considering the clinical translation of protein microarray–based approaches.

The most pervasive limitation is the dependence on antibody quality and specificity. Many studies rely on commercial antibody arrays, but batch-to-batch variability, cross-reactivity, and lack of standardized validation protocols remain substantial concerns (82, 133). While sandwich-based assays offer improved specificity through dual-antibody recognition, they are constrained by the availability of well-matched antibody pairs and are less scalable for ultra-high-plex applications. Label-based assays, although more scalable, suffer from variability in labeling efficiency across different proteins, which can compromise quantitative accuracy (71, 72).

A second limitation is the predominantly retrospective and case-control design of most included studies. Many biomarker discovery studies cited in this review (27, 86, 87) employed relatively modest sample sizes and lacked independent, multi-center prospective validation. This raises concerns about overfitting and limits the generalizability of reported diagnostic algorithms. For instance, while Ahn et al. (87) reported >85% accuracy in training and test sets, the performance of such multi-protein signatures in real-world screening populations—which have much lower disease prevalence—remains to be determined.

Third, most protein microarray studies in GC have focused on a restricted set of predefined targets (e.g., cytokines, growth factors, MMPs). Although this targeted nature is advantageous for hypothesis-driven research and for focusing on biologically relevant pathways, it precludes unbiased discovery of novel protein signatures. As a result, the field may have missed unexpected mediators that could be identified through untargeted mass spectrometry–based approaches. Indeed, the complementary use of MS for discovery followed by protein microarray for validation—exemplified by Cui et al. (32) represents a more balanced strategy that mitigates this limitation.

Fourth, technical heterogeneity across studies, including differences in array platforms (FPPA vs. RPPA), detection methods (sandwich vs. label-based), sample types (serum, plasma, tissue lysates, urine), and data normalization strategies—complicates cross-study comparison and meta-analysis. The lack of standardized reporting guidelines for protein microarray experiments in GC research further exacerbates this issue.

Finally, although reverse-phase protein arrays (RPPAs) offer the advantage of sample-centric profiling and are well suited for large-scale cohort studies (73, 75), their application in GC has been relatively limited compared to forward-phase arrays. This may be due to challenges in antibody validation and the absence of molecular weight separation, which increases the risk of off-target binding (73).

### 4.3 Integration into current understanding and advancement of prevailing views

The findings summarized in this review collectively advance the current understanding of GC biology in several important ways.

First, they reinforce the concept that GC is not merely a disease of epithelial cell–autonomous alterations but rather a complex ecosystem shaped by dynamic interactions among cancer cells, stromal fibroblasts, immune cells, endothelial cells, and the extracellular matrix. Protein microarray studies have provided direct, quantitative evidence for specific ligand–receptor pairs (e.g., IL-8–CXCR1/2, CCL2–ACKR1, CCL5–CCR5) that mediate crosstalk between compartments (96, 105, 118). This systems-level view complements the molecular subtypes defined by The Cancer Genome Atlas (TCGA) (39) and Asian Cancer Research Group (ACRG) (40) by adding a functional, proteomic layer that captures intercellular communication networks.

Second, the shift from single biomarkers to multi-protein signatures has challenged the traditional reductionist approach to cancer diagnosis. The consistent observation that combinatorial panels outperform individual markers—even when individual markers show modest performance (92)—supports a probabilistic, network-based framework for interpreting circulating proteomic data. This aligns with emerging views in precision oncology that disease states are better represented by patterns of analytes rather than by any single “magic bullet” biomarker.

Third, protein microarray studies have uncovered non-canonical mechanisms of therapy resistance that extend beyond classical drug efflux or target mutation. For example, the identification of a CCL2–SQSTM1 positive feedback loop that suppresses pro-apoptotic autophagy (115) reveals a novel connection between chemokine signaling and autophagic flux, suggesting that resistance can arise from adaptive rewiring of cell survival pathways. Similarly, the finding that DARPP-32 enhances gefitinib resistance by stabilizing EGFR and promoting ERBB3 interaction (122) provides a mechanistic explanation for resistance in tumors without canonical EGFR mutations.

Fourth, the application of protein microarrays to liquid biopsy–type samples (serum, plasma, urine) has advanced the view that minimally invasive, longitudinal monitoring of GC is feasible. The demonstration that surgical resection leads to a uniform drop in multiple plasma cytokines (91) and that urinary CXCL2 levels correlate with peritoneal metastasis (108) supports the use of protein signatures for dynamic treatment response assessment and early detection of recurrence.

This stepwise translational pipeline—from discovery-based screening and candidate verification to mechanistic investigation and clinical validation—highlights the role of protein microarrays in bridging biomarker discovery with clinical applications in diagnosis, prognosis, and therapy for gastric cancer.

### 4.4 Concluding remarks for the discussion

In summary, protein microarray technologies have matured from descriptive discovery tools to functional platforms capable of uncovering mechanistic insights and guiding therapeutic decisions in gastric cancer. The transition from single biomarkers to multi-protein signatures, the integration of proteomic data with complementary omics layers, and the application of artificial intelligence for pattern recognition collectively hold promise for advancing precision oncology. However, realizing this promise will require addressing current limitations related to standardization, prospective validation, and technological innovation, with the goal of translating protein microarray–based discoveries into clinically actionable tools that improve early detection, prognostic stratification, and therapeutic monitoring for patients with gastric cancer.

## 5 Conclusion

Protein microarray based proteomic technologies have emerged as versatile and powerful tools in gastric cancer (GC) research, offering an effective balance between throughput, sensitivity, and functional insight. By enabling the simultaneous analysis of hundreds to thousands of proteins and their post-translational modifications, these platforms provide a systems-level perspective that complement genomic and transcriptomic approaches. Substantial progress has been made in leveraging protein microarrays for biomarker discovery, early detection, prognostic evaluation, and molecular subtyping. In particular, the shift from single biomarkers to multi-protein signatures has significantly enhanced diagnostic performance and highlighted their potential for clinical application.

Beyond biomarker identification, protein microarrays have deepened our understanding of the complex signaling networks and tumor microenvironment interactions that drive GC progression, including pathways related to immune regulation, angiogenesis, and therapeutic resistance. Their application in drug response assessment and longitudinal monitoring further underscores their value in guiding precision treatment strategies. When integrated with complementary proteomic platforms such as mass spectrometry and emerging affinity-based technologies, protein microarrays serve as a critical bridge between discovery-phase research and clinical validation.

Despite ongoing challenges related to antibody quality, standardization, and reproducibility, continued technological advancements and multi-omics integration are expected to accelerate clinical translation. The future clinical value of protein microarrays in gastric cancer will depend less on identifying individual markers and more on developing reproducible, biologically interpretable, multi-protein signatures that can be validated across cohorts and integrated with endoscopic, histopathological, genomic, and clinical data. Collectively, protein microarrays are poised to play an increasingly central role in advancing precision oncology and improving clinical outcomes in gastric cancer.
